# Awe promotes moral expansiveness *via* the small-self

**DOI:** 10.3389/fpsyg.2023.1097627

**Published:** 2023-03-06

**Authors:** Ji Young Song, Christoph Klebl, Brock Bastian

**Affiliations:** ^1^Melbourne School of Psychological Sciences, The University of Melbourne, Melbourne, VIC, Australia; ^2^School of Psychology, University of Queensland, Brisbane, QLD, Australia

**Keywords:** awe, moral expansiveness, moral concern, small-self, vastness vis-a-vis the self, connectedness, Virtual Reality

## Abstract

The experience of awe has been shown to challenge how people think about themselves and the world around them, linking them to something greater than themselves. We investigated whether this emotional experience of awe may also challenge the boundaries of our moral consideration, leading to a generalized expansion in our moral worlds. Across five studies (*N* = 990), we examined whether awe might promote *moral expansiveness*; that is, increased moral concern across a broad range of entities (e.g., out-groups, animals, plants, environments). Cross-sectional Studies 1a and 1b, found dispositional awe was related to greater moral expansiveness. Experimental Studies 2 and 3, using video-induced awe, found consistent indirect effects on moral expansiveness, *via* self-reported awe and the small-self sense of vastness. Experimental Study 4, using Virtual Reality induced awe, found those in the awe condition (vs. control) reported greater moral expansiveness, and this was fully mediated by the small-self sense of vastness. Our findings show awe expands our sense of connectedness to the broader world, and through this, increases the breath of our moral concern.

## Introduction

*“Then I was standing on the highest mountain of them all, and round about beneath me was the whole hoop of the world. And while I stood there, I saw more than I can tell and I understood more than I saw; for I saw seeing in a sacred manner the shape of all things in the spirit, and the shape of all shapes as they must live together like one being. And I saw that the sacred hoop of my people was one of many hoops that made one circle.”*

(Black Elk as quoted by Neihardt, 2014, p. 26)

The journey up the great mountains of the world often leaves us awe-struck, as the sight of vast horizons reminds us that we are part of a much larger natural world. These kinds of awe experiences, and others like them, may not just broaden our awareness of the physical world but may also expand our moral worlds too. One of the key psychological processes through which awe may have this type of effect is *via* its impact on the self-concept (Shiota et al., 2007; Piff et al., 2015; Yang et al., 2016; Wang and Lyu, 2019). Specifically, awe has been found to activate a sense of small-self, through which people feel humbler in the grand scheme of things (self-diminishment) and more connected to the greater whole (vastness vis-à-vis the self; Piff et al., 2015). By shaping how the self is experienced in relation to one’s environment, and increasing a sense of generalized connection, the experience of awe may also lead to a generalized extension of moral consideration for others. Here, we examine whether the experience of awe promotes a more morally expansive orientation (Crimston et al., 2016), leading to an increase in moral concern across a broad range of targets.

### Awe and prosociality

Awe is defined as an emotional experience that arises through an encounter with something that transcends one’s ordinary day-to-day experience, thereby challenging how a person thinks about themselves and the world around them and incorporating both a sense of vastness and a need for accommodation (Keltner and Haidt, 2003). Research has linked the experience of awe to pro-social responses, such as generosity in economic and lottery games, donating money and volunteering time to help others (Rudd et al., 2012; Piff et al., 2015; Prade and Saroglou, 2016; Guan et al., 2019). It has also been linked to environmentally conscious behavioral intentions (Zhao et al., 2018; Wang and Lyu, 2019). Notably, however, some research has found experiences of awe which may feel threatening, can decrease pro-social responses to others in those with low self-esteem (Hornsey et al., 2018).

Awe’s relationship with prosocial behavior and moral concern appears to be driven, at least in part, by changes in the self-concept. After experiencing awe people frequently report a small-self, which is defined by the two key components; ‘self-diminishment’ and ‘vastness vis-à-vis the self’ (Shiota et al., 2007; Piff et al., 2015). The ‘self-diminishment’ component of the small-self captures the way awe often makes one feel smaller in comparison to something larger (e.g., “I feel insignificant in the grand scheme things of things”; Piff et al., 2015). Studies have found this effect of awe is linked to a greater sense of humility (Stellar et al., 2018) and appears to be a key factor in promoting generosity toward others (Piff et al., 2015). The small-self sense of ‘vastness vis-à-vis the self’ captures the way that being in presence of vast awe-evoking stimuli (e.g., “I am in the presence of something greater”), one also often feels a greater sense of connectedness to a greater whole (e.g., “I feel part of a greater whole”; Piff et al., 2015). That is, awe often makes us feel a sense of connectedness to a larger world than we might ordinarily feel in everyday life. For instance, Shiota et al. (2007) found people in awe of a Tyrannosaurus Rex fossil (vs. controls) were more likely to feel part of larger super-ordinate categories, such as all humanity or the Earth. And this sense of enlarged connectedness appears to feature in the most intense awe experiences, with qualitative work examining Astronaut’s experiences of the Overview Effect (seeing the Earth from near space) reporting consistent themes of feeling not only a sense of profound awe, but also an awakened sense of global unity with all peoples and the Earth as a whole (White, 2014; Yaden et al., 2016).

Further, recent work using related measures of connectedness suggests the small-self sense of ‘vastness vis-à-vis the self’ may also be a pathway to broader moral concern. For instance, Luo et al. (2022) found recalling awe-inspiring stories of awe from the COVID-19 pandemic (e.g., emergency workers fighting in tough situations; vs. recalling amusing/neutral memories) promoted a sense of connection to all living things (e.g., “I experienced a sense of oneness with all things”), which in turn mediated a range of prosocial measures to help others; including helping foreign countries with COVID-19 support, willingness to donate blood, and register as a blood donor. Similarly, Pizarro et al. (2021) found eliciting awe through videos (e.g., Mongolian horse riders) was related to a greater sense of connectedness to people in general, which in turn mediated a greater willingness to collectively help others (e.g., collaborating with an NGO to help a humanitarian cause). These relationships appear to be ecologically robust, Goldy et al. (2022) examining tweets during the 2017 Solar Eclipse found people in the path of totality (vs. people living outside the path of the eclipse) were more likely to use affiliative, collective, and pro-social language.

### Awe and moral expansiveness

Experiencing awe appears to increase specific prosocial intentions and behavior, but whether it may have a broader and moral generalized impact on how people think about their moral worlds, and specifically the size and composition of those worlds, remains unexplored. The possibility that awe may have highly generalized effects is supported by evidence that its impact on cognition, emotion, and behavior occurs *via* shifting how people experience their sense of self (i.e., *via* the small self). In this way, awe has the capacity to change how people view the world in a rather general way, and this may influence how, and to whom, they extend their moral consideration and concern.

Moral expansiveness is a construct which aims to capture both the extent (number of entities) and depth (level of felt obligation or concern for specific entities) of a person’s felt moral concern for a broad range of entities, and therefore the size of their moral worlds (Crimston et al., 2016, 2018a). While current work on moral expansiveness has focused on individual differences in the size of a person’s moral world as associated with universal values or pro-social behavior (Crimston et al., 2016), or its relationship to relatively stable socio-ecological factors (e.g., income inequality; Kirkland et al., 2022), less work has focused on situational factors that may lead to changes in the expansiveness of a person’s moral world.

Related research suggests that cognitive framing (such as exclusionary vs. inclusive mindsets; Laham, 2009) or different comparison sets (such as comparing how similar animals are to humans vs. comparing how similar humans are to animals; Bastian et al., 2012) can lead to generalized differences in the size of a person’s moral circle. To date, however, no research has examined emotional pathways toward greater moral expansiveness. As noted by Haidt (2001, 2003), moral judgments are often influenced by emotional processes. Consistent with this, there is some evidence that trait levels of moral expansiveness are associated with the dispositional tendency to feel greater empathy (Crimston et al., 2016) or compassion (Crimston et al., 2022), however such emotional states are directly related to the capacity to feel concern for others. Whether the experience of awe may also lead to higher levels of moral expansiveness, not through the capacity to feel concern, but through a general shift in how people experience their self-concept and their relationship to the broader environment – that is, through the tendency to feel connected to a greater whole – has not been explored. We suggest that awe may play an important role in expanding a person’s moral world.

### The present research

In the current research, we examined whether awe may promote a morally expansive orientation to the world. Furthermore, whether this relationship can be explained by awe-associated changes in a person’s experience of their self-concept – referred to as the small self. We examined this possibility across five studies. In Studies 1a and 1b, we examined the relationship at the trait level between dispositional awe and individual differences in moral expansiveness. In follow-up experiments, we examined whether state experiences of awe shift people toward greater moral expansiveness. In Experiments 2 and 3, awe was induced using videos of the destructive power of mother nature (negatively-valanced nature awe), the otherworldliness of water-droplets in extreme slow-motion (non-nature awe), and the majestic beauty of nature (positively-valanced nature awe). In Experiment 4, awe was induced using a Virtual Reality (VR) simulation of the overview effect (White, 2014; Yaden et al., 2016), in which people felt that they were astronauts seeing the Earth from near space.

## Studies 1a and 1b: Dispositional awe and moral expansiveness

In Studies 1a (United States) and 1b (Australia), we examined the relationship between awe and moral expansiveness at the trait level. The dispositional tendency to experience greater awe in daily life has been found to show similar effects as situationally triggered state experiences of awe. For instance, studies have found both the dispositional tendency and the state experience of awe to be related to greater pro-sociality (Piff et al., 2015), environmentalism (Zhao et al., 2018), and humility (Stellar et al., 2018). We used the disposition for awe subscale from the Disposition for Positive Emotions scale (Shiota et al., 2006) to examine whether the dispositional tendency to feel awe is related to greater levels of moral expansiveness. In Study 1b, we also included measures for the disposition for joy, contentment, pride, love, compassion, and amusement in order to control for a general disposition for positive emotions, thereby allowing us to test whether any observed effects were specific to awe.

## Method

### Participants

#### Study 1a (United States)

An initial power analysis indicated that a sample size of 193 would be required to detect a small effect size (*r* = .20) at *α* = .05 and a power of 0.80 (Faul et al., 2009). To account for exclusion of participants who fail attention checks, we over-recruited an initial sample of 210 participants living in America recruited through Amazon Mechanical Turk (MTurk). Thirteen participants were excluded for failing attention checks.^1^ Among the final sample (*N* = 197), 57.9% were men and 40.6% women; ages ranged between 20 and 69 (*M_age_* = 38.10, *SD* = 11.37); 77.2% were White or European, 9.6% Black or African American, 4.6% Latino or Hispanic, and 3% Native American, 2% Asian. Participants were compensated US$2.50.

#### Study 1b (Australia)

Study 1b, aimed to replicate the findings of Study 1a and control for a range of other positive emotions. A power analysis for linear multiple regressions using conservative estimation of a small effect size (*f*^2^ = .05), *α* = .05, power = .80, and controlling for 9 predictors indicated that a sample size of 322 participants would be required. Based on this, we over-recruited a sample of 363 students enrolled in a first-year psychology course at an Australian university, who participated in exchange for course credit. However, 71 students were excluded for failing attention checks and a further four participants withdrew their consent at the end of the study.^2^ This provided a final sample of 288 students with a mean age of 19.54 years (*SD* = 3.27); 206 were women (71.5%), 73 men (25.3%), and four were non-binary or third gender people (1.4%); 166 identified as Asian (57.6%), 99 as White or European (34.4%), ten as Arab or Middle Eastern (3.5%), two as Black or African (0.7%), and one as Latino or Hispanic (0.7%).

#### Materials and procedures

Both Studies 1a and 1b were administered as a Qualtrics online survey, which were part of larger studies examining moral and social identity. After giving consent, participants first completed a series of online personality questionnaires. This included the Disposition for Awe subscale (e.g., “I often feel awe”; Study 1a, *α* = .85; Study 1b, *α* = .80) from the Dispositional Positive Emotions Scale (DPES; Shiota et al., 2006). Participants were asked to what extent they agreed with statements, and items were answered on a 7-point Likert scale (1 = *strongly disagree*, 7 = *strongly agree*). This scale has been widely employed as a measure of people’s tendency to experience awe in their day-to-day lives (e.g., Stellar et al., 2017; Zhao et al., 2019).

In Study 1b, participants completed the full DPES (Shiota et al., 2006). This included the subscales for Disposition for Joy (6-item; e.g., “I often feel bursts of joy”; *α* = .82), Contentment (5-item; e.g., “I am at peace with my life”; *α* = .86), Pride (5-item; e.g., “Many people respect me”; *α* = .79), Love (6-item; e.g., “I develop strong feelings of closeness to people easily”; *α* = .83), Compassion (5-item; e.g., “It’s important to take care of people who are vulnerable”; *α* = .83), and Amusement (5-item; e.g., “I make jokes about everything”; *α* = .79).

In both Studies 1a and 1b, to assess their level of moral expansiveness, participants next completed the Moral Expansiveness short form (MESx; Crimston et al., 2018b; Study 1a, *α* = .58; Study 1b, *α* = .76). The MESx has shown convergent validity with the original Moral Expansiveness Scale (Crimston et al., 2016), and is psychometrically valid and reliable (Crimston et al., 2018b). The MESx prompts participants to consider the moral standing of ten entities, ranging from close targets (e.g., family member, citizen of your country) to a range of distal targets (e.g., mentally challenged individual, dolphin, old-growth forest). Participants indicate the degree of moral standing of these entities by placing them within four moral boundaries: the inner circle (“highest level of moral concern and standing… moral obligation to ensure their welfare and a sense of personal responsibility for their treatment”; scored 3), the outer circle (“moderate moral concern and consideration…”; scored 2), the fringes of moral concern (“minimal moral concern and standing…”; scored 1), and outside the moral boundary (“no moral concern or standing…”; scored 0). Participants overall level of moral expansiveness was calculated by averaging scores across all 10 entities.

Participants next completed a set of demographic items that included single-item measures of their economic conservatism (“Please indicate your political beliefs from left/progressive to right/conservative on issues of the economy, e.g., social welfare, government spending, tax cuts.”; 1 = *left/progressive*, 7 = *right/conservative*), social conservatism (“Please indicate your political beliefs from left/progressive to right/conservative on social issues, e.g., immigration, same-sex marriage, abortion.”; 1 = *left/progressive*, 7 = *right/conservative*), and religiosity (“If you do follow a religion, how important is that religion in your daily life?”; 0 = *Not applicable*, 1 = *Not at all important*, 7 = *Extremely important*). Finally, self-perceived social status was measured using the MacArthur Scale of Subjective Social Status (Adler et al., 2000) which asked participants to indicate where they saw themselves on ladder representing the social hierarchy (Rung 1 = *bottom of society*, Rung 10 = *top of society*).

### Results and discussion

In line with our main hypothesis, across both Studies 1a and 1b, we found a tendency to experience awe in life was associated with increased moral expansiveness (Table 1). Further, using linear regressions, in Study 1a, we found disposition for awe (*β* = .30, *p* < .001) significantly predicted moral expansiveness while controlling for demographic variables (political orientation, religiosity, social status). In Study 1b, we found disposition for awe (*β* = .16, *p* = .024) significantly predicted moral expansiveness when controlling for both demographics (political orientation, religiosity, social status) and disposition for other positive emotions (joy, contentment, pride, love, and amusement). While this generally demonstrated that the relationship between awe and moral expansiveness was not explained by general positivity, we did find when controlling for disposition for compassion, the disposition for awe (*β* = .12, *p* = .081) no longer predicted moral expansiveness. This may be because compassion is closely aligned with moral concern for others (see Shiota et al., 2006), and was a stronger predictor of moral expansiveness, *r*(281) = .23, *p* < .001, compared to dispositional awe, *r*(281) = .17, *p* = .006. A full table of correlations and linear regressions are reported in the Supplementary material.

**Table 1:** Zero-order correlations between disposition for awe, moral expansiveness, and demographic variables in Studies 1a and 1b.

Variable	1	2	3	4	5	6
Study 1a (United States)						
1. Disp. Awe^a^	–					
2. Moral Exp.^b^	.26***	–				
3. Religiosity	.23**	.23**	–			
4. Eco. Conserv.^c^	.15*	−.15*	.57***	–		
5. Soc. Conserv.^d^	.20**	−.12	.60***	.87***	–	
6. Social Status^e^	.28***	−.03	.38***	.42***	.41***	–
Mean	4.89	1.66	3.80	3.94	3.71	5.47
*SD*	1.15	.41	2.75	2.00	2.09	2.29
Study 1b (Australia)						
1. Disp. Awe^a^	–					
2. Moral Exp.^b^	.17**	–				
3. Religiosity	.07	.05	–			
4. Eco. Conserv.^c^	.01	−.16**	.16*	–		
5. Soc. Conserv.^d^	−.01	−.14*	.26***	.53***	–	
6. Social Status^e^	.16**	.00	−.04	.00	−.07	–
Mean	4.78	1.76	2.38	3.23	2.59	6.66
*SD*	1.02	.44	2.23	1.34	1.53	1.97

*Note*. Study 1a *N* = 195–199; Study 1b *N* = 269–288. ^a^ Disposition for awe. ^b^ Moral expansiveness. ^c^ Economic conservatism. ^d^ Social conservatism. ^e^ Self-perceived social status. ***p < .001, **p < .01, *p < .05.

The results of Studies 1a and 1b are consistent with our prediction that experiences of awe are associated with increased moral expansiveness. Furthermore, this relationship remained robust when controlling for political orientations, religiosity, and a general disposition for other positive emotions.

## Study 2: The experience of awe and moral expansiveness

Building on the trait level findings in Studies 1a and 1b, we examined the causal relationship between awe and moral expansiveness by experimentally inducing states of awe using videos. The experiment closely replicated previous methods used to induce awe by Piff et al. (2015) and used videos showing negatively-valanced nature and non-nature awe.

### Method

#### Participants

Power-analysis, assuming medium effect size (*f* = .25) and setting power at .80, indicated a one-way ANOVA would require 159 participants to detect a main effect between three conditions. Based on this, we recruited an initial sample of 186 psychology students enrolled at an Australian university were recruited in exchange for course credit. Nineteen participants were excluded for failing attention checks.^3^ The final sample (*N* = 167) comprised 127 women (76%), 39 men (23.4%), and one non-binary person (.6%); aged 17 to 57 years of age (*M_age_* = 20.20, *SD* = 4.93); 89 identified as Asian (53.3%), 52 as White or European (31.1%), six as Middle Eastern or Arab (3.6%), one as South American (0.6%), and one as Aboriginal or Torres Strait Islander (0.6%).

#### Materials and procedure

Participants were brought into the lab, after giving consent they first completed a series of personality survey that were part of a larger project examining moral psychology and the self.

Participants were then randomly assigned to one of three video conditions; namely, a negatively-valanced nature awe, non-nature awe, or control video condition. In the negatively-valanced nature awe condition, participants watched a 3-min video montage of the overwhelming power and destructive force of mother nature (e.g., tornadoes, hurricanes)^4^. In the non-nature awe condition, participants watched a slow-motion video of colored water droplets colliding.^5^ In the control condition, participants watched a DIY video of a man making a wooden countertop.^6^ These three videos were identical to those used previously by Piff et al. (2015) to induce awe and neutral emotional states.

After watching the video, participants next completed an emotion manipulation check. In random order, they were asked to what extent they had felt 10 different emotions during the video they just watched: awe, amusement, anger, anxiety, disgust, fear, nervousness, pride, sadness, and happiness. Responses were taken on a 7-point Likert scale (1 = *not at all*, 7 = *extremely*).

Participant’s sense of small-self was next measured using the 10-item small-self scale (*α* = .85; Piff et al., 2015). The small-self scale measured both ‘vastness vis-à-vis the self’ (e.g., “I feel part of a greater whole”; *α* = .87) and self-diminishment (e.g., “I feel small or insignificant”; *α* = .81). Items were randomized and on a 7-point Likert scale (1 = *strongly disagree*, 7 = *strongly agree*).

Next, participants completed a modified moral expansiveness scale (MES; Crimston et al., 2016). The modified-MES (*α* = .95) used the same 10 categories of entities (30 in total) from the original MES: family/friends (e.g., family member; *α* = .64), in-group (e.g., Australian citizen; *α* = .86), out-group (e.g., member of opposing political party; *α* = .80), revered (e.g., Australian soldier; *α* = .79), stigmatized (e.g., refugee; *α* = .79), villains (e.g., terrorist; *α* = .80), high-sentient animals (e.g., Chimpanzee; *α* = .89), low-sentient animals (e.g., Chicken; *α* = .88), plants (e.g., redwood tree; *α* = .91), and environment (e.g., old-growth forest; *α* = .87). However, the modified-MES differed from the original MES in several ways. First, the prompt was reworded to emphasize how much moral concern participants *feel* for entities, rather than the original prompt which emphasized *moral obligations* and *responsibilities*. Second, participants were asked how much moral concern they felt for each entity separately on a Likert scale (1 = *no moral concern*, 7 = *extreme moral concern*). Both these changes were aimed at retaining the previously validated set of entities to measure moral expansiveness, while better tapping into people’s intuitive and felt moral concern for each entity in the moment.

Next, participants completed a final set of demographics which included the same items measuring social conservatism, economic conservatism, and religiosity as Studies 1a and 1b.

### Results

We first examined whether there were differences in self-reported awe between conditions. A one-way analysis of variance (ANOVA) indicated there was a significant difference between conditions in self-reported awe, *F*(2,163) = 34.62, *p* < .001, *η*_p_^2^ = .30. *Post-hoc* Bonferroni revealed participants in the negative-nature awe condition experienced greater awe compared to those in the control condition, *p* < .001, 95% CI [1.80, 3.43], but not compared to those in the non-nature awe condition, *p* = 1.00, 95% CI [−0.57, 1.15]. And participants in the non-nature awe condition experienced greater awe compared to those in the control condition, *p* < .001, 95% CI [1.44, 3.20] (see Table 2). Mean scores for all self-reported emotional states are reported in the Supplementary material.

**Table 2:** Mean and standard deviation scores for self-reported awe, small-self, and moral expansiveness in Studies 2, 3, and 4.

	Study 2 (Videos)			Study 3 (Videos)		Study 4 (Virtual Reality)	
	Neg. Nat. Awe^a^	Non-nat. Awe^b^	Control	Pos. Nat. Awe^c^	Control	VR Awe^d^	Control
	(*n* = 63)	(*n* = 47)	(*n* = 57)	(*n* = 93)	(*n* = 120)	(*n* = 62)	(*n* = 63)
Awe^e^	5.02 (1.73)^ij^	4.72 (2.11)^j^	2.40 (1.70)	5.20 (1.89)^j^	2.58 (1.71)	5.58 (1.72)^j^	2.60 (1.66)
Small-self	5.13 (0.88)^ij^	4.57 (0.73)	4.30 (0.97)	4.47 (1.43)	4.12 (1.32)	5.18 (0.96)^j^	4.55 (0.78)
Vastness^f^	5.22 (0.96)^ij^	4.78 (1.14)	4.33 (1.22)	4.54 (1.66)	4.17 (1.73)	5.48 (1.10)^j^	4.58 (1.03)
Self-diminish^g^	5.03 (1.08)^ij^	4.37 (0.85)	4.27 (1.17)	4.41 (1.57)	4.06 (1.43)	4.88 (1.10)	4.52 (0.98)
Moral Exp^h^	4.60 (0.90)	4.43 (0.88)	4.33 (1.03)	4.55 (1.26)	4.39 (1.12)	4.94 (1.05)^j^	4.34 (1.33)

*Note*. ^a^ Negative nature awe. ^b^ Non-nature awe. ^c^ Positive nature awe. ^d^ Virtual Reality awe. ^e^ Self-reported awe. ^f^ Vastness vis-à-vis the self. ^g^ Self-diminishment. ^h^ Moral expansiveness. ^i^ These means are significantly greater than those in non-nature awe condition, *p*s < .05. ^j^ These means are significantly greater than those in the control condition, *p*s < .05.

We next examined whether participants in the awe conditions experienced a smaller self (Table 2). This revealed there were significant differences in small-self across conditions, *F*(2, 164) = 13.94, *p* < .001, *η*_p_^2^ = .15. *Post-hoc* Bonferroni analysis found participants in the negative nature awe condition experienced a smaller sense of self than participants in the non-nature awe, *p* = .004, 95% CI [0.15, 0.96], and the control conditions, *p* < .001, 95% CI [0.44, 1.22].

We next examined our main hypothesis that the experience of awe would lead to greater moral expansiveness. An initial ANOVA indicated no significant difference in moral expansiveness between conditions (Table 2), *F*(2, 164) = 1.29, *p* = .278, *η*_p_^2^ = .02. Given Hayes (2009) and Rucker et al. (2011) have demonstrated that indirect effects may still be present in the absence of a main effect, we examined the theoretically predicted indirect pathways between conditions (awe vs. control) and moral expansiveness through self-reported awe. We first contrast coded the awe and control conditions (coded as negative awe = 1, non-nature awe = 1, and control = −2). We then followed Hayes (2018) bootstrapping procedures, using Model 4 in the SPSS PROCESS macros with 5,000 iterations, which found a significant indirect pathway between conditions (awe contrast to control) and moral expansiveness and self-reported awe, *B* = .08, *SE* = .04, 95% CI [0.01, 0.15].

**Figure 1:**
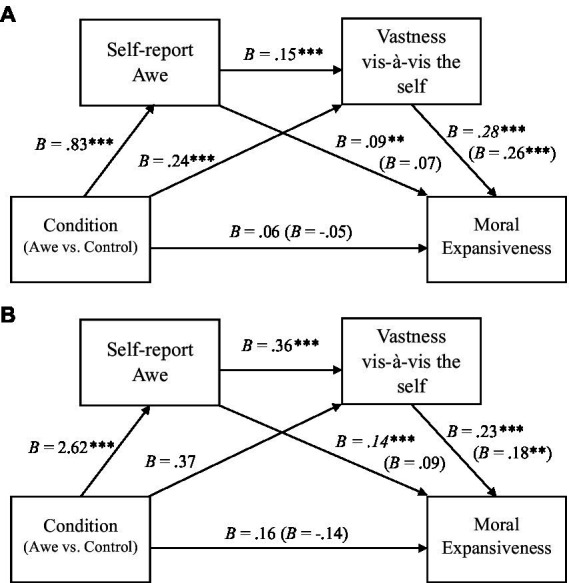
Indirect pathway models in Studies 2 and 3. Panels **A** (Study 2) and **B** (Study 3) presents the serial mediation model of conditions (awe vs. control) to moral expansiveness, *via* first self-report awe and then ‘vastness vis-à-vis the self.’ In Panel **A**, condition is contrast coded (negative awe = 1, non-nature awe = 1, control = −2). In Panel **B**, condition is a binary (control = 0, awe = 1). Unstandardized coefficients are presented. Numbers in parentheses indicate coefficients when all variables are predicting moral expansiveness. *p < .05, **p < .01, ***p < .001.

While we did not find a main effect of condition (awe vs. control) on moral expansiveness, as an exploratory analysis we examined the role of the small-self by testing a serial mediation model in which condition (awe vs. control) predicted moral expansiveness, first through awe and then the small-self. Following Hayes (2018) bootstrapping procedures, and using Model 6 in the SPSS PROCESS macro with 5,000 iterations, we found a significant indirect pathway through which conditions (awe contrast to control) promoted greater levels of moral expansiveness, first through self-reported awe, and then the small-self, *B* = .01, *SE* = .03 95% CI = [0.002, 0.04]. We next examined the small-self components of ‘vastness vis-à-vis the self’ and ‘self-diminishment’ separately. Using the same bootstrapping procedure, this revealed a significant indirect pathway first through awe and then ‘vastness vis-à-vis the self’ (Figure 1), *B* = .02, *SE* = .01, 95% CI [0.002, 0.05], but not through awe and then ‘self-diminishment,’ *B* = .00, *SE* = .01, 95% CI [−0.01, 0.01]. This was also consistent with hierarchical linear regression modeling that found ‘vastness vis-à-vis the self’ was a significant predictor of moral expansiveness, while controlling for religiosity and political orientations, whereas ‘self-diminishment’ was found to be non-significant predictor of moral expansiveness (see Table 3).

**Table 3:** Hierarchical linear regression of condition (awe vs. control), awe, and small-self (‘vastness vis-à-vis the self’ and self-diminishment) predicting levels of moral expansiveness in Studies 2 and 3.

Step	Predictor	*B*	*SE*	*β*	*p*	*R* ^2^	*F*	*p*
Study 2 (Australia)								
1						.06	4.86	.009
	Condition^a^	−.02	.06	−.03	.711			
	Awe^b^	.11	.04	.26	.006			
2						.21	5.72	<.001
	Condition	−.06	.06	−.08	.358			
	Awe	.07	.04	.17	.061			
	Small-self							
	Vastness^c^	.30	.07	.37	< .001			
	Self-dim^d^	−.08	.07	−.09	.246			
	Religiosity	.03	.03	.07	.399			
	Eco. Cons.^e^	−.02	.07	−.03	.791			
	Soc. Cons.^f^	−.13	.06	−.212	.026			
Study 3 (United States)								
1						.06	4.86	.009
	Condition^g^	−.07	.18	−.03	.711			
	Awe	.11	.04	.26	.006			
2						.21	5.72	<.001
	Condition	−.16	.17	−.08	.358			
	Awe	.07	.04	.17	.061			
	Small-self							
	Vastness	.30	.07	.37	<.001			
	Self-dim	−.08	.07	−.09	.246			
	Religiosity	.03	.03	.07	.399			
	Eco. Cons.	−.02	.07	−.03	.791			
	Soc. Cons.	−.13	.06	−.212	.026			

*Note*. Study 2, *N* = 158. Study 3, *N* = 208. B = Unstandardized coefficients. SE = Standard Error of B. *β* = Standardized coefficients. ^a^ Study 2 condition contrast coded (negative awe = 1, non-nature awe = 1, control = −2). ^b^ Self-report awe. ^c^ ‘Vastness vis-à-vis the self.’ ^d^ Self-diminishment. ^e^ Economic conservatism. ^f^ Social conservatism. ^g^ Study 3 condition is binary (control = 0, awe = 1).

### Discussion

While Study 2 failed to detect a main effect of condition (awe vs. control) on moral expansiveness, the presence of a significant indirect pathway between condition (awe vs. control) and moral expansiveness, through self-reported awe, was supportive of our main hypothesis. That is, insofar as participants in the conditions (awe vs. control) experienced a stronger sense of awe, they also reported greater moral expansiveness. Furthermore, the indirect pathway analyses were supportive of our secondary hypothesis that small-self would be the mechanism through which awe promoted moral expansiveness. However, while significant indirect pathways may be consistent with a causal mechanism (Hayes, 2009), it is difficult to rule out unknown third variables without a direct effect of experimental condition (Rohrer, 2019).

## Study 3: Nature-induced awe and moral expansiveness

To address the failure to detect a main effect in Study 2, which induced awe using videos of the destructive power of nature (negatively-valanced nature awe) and slow-motion water droplets colliding (non-nature awe), in Study 3 we induced awe using a video of the wonder and beauty of nature (positively-valanced nature awe). We reasoned that awe experienced through the positive side of nature may have a more powerful effect on moral expansiveness, as the wonder and beauty of nature has been found to be one of the most commonly reported sources of awe in people’s lives (Bai et al., 2017; Gordon et al., 2017) and similar video inductions have been widely used to induce awe and promote a range of prosocial effects (e.g., Piff et al., 2015; Wang and Lyu, 2019).

### Method

#### Participants

Power analysis, assuming a small-effect size (*f* = .20) and power of 0.80, indicated a sample size of 200 would be needed to detected a main effect in a one-way ANOVA between two conditions. Based on this, we over-recruited an initial sample of 222 participants living in the United States through MTurk. Nine participants were excluded for failing attention checks.^7^ The final sample (*N* = 213) contained 110 men (51.6%), 97 women (45.5%), and 1 non-binary person (0.5%); with ages ranging between 21 and 67 years of age (*M_age_* = 39.47; *SD* = 10.81). Participants were compensated US$1.

#### Materials and procedure

The study was administered through an online survey on Qualtrics. After giving consent, participants first completed a set of personality questionnaires that were part of a larger study investigating the self and moral psychology. Participants were then randomly assigned to either an awe or control condition. In the awe condition, participants viewed a video montage of sweeping forests and grand mountain vistas, from the BBC’s *Planet Earth* documentary series.^8^ This kind of nature montage has been widely used in the awe literature to elicit states of positively valanced awe (e.g., Valdesolo and Graham, 2014; Piff et al., 2015; Zhao et al., 2018). In the control condition, participants viewed a video of a man in a forest explaining the use of trail markers.^9^ The control condition was designed to control for exposure to nature but avoiding any elicitation of awe.

Following Study 2, participants then completed the same manipulation check measuring emotional states (e.g., awe, amusement), followed by the small-self scale (Piff et al., 2015; *α* = .90), and modified-MES (modified version of MES; Crimston et al., 2016; *α* = .96). Next, participants completed a final set of demographics which included the same items measuring social conservatism, economic conservatism, and religiosity as Studies 1a and 1b.

### Results

As predicted, participants in the awe condition reported experiencing significantly higher levels of awe compared to those in the control condition (Table 2), *t*(211) = 10.63, *p* < .001, *d* = 1.45, 95% CI [2.14, 3.12]. Unexpectedly, however, there was no significant effect of condition on the small-self, *t*(211) = 1.90, *p* = .059, *d* = .25, 95% CI [−0.01, 0.73]. When examining the small-self subscales, neither were significantly different across the two conditions; ‘vastness vis-à-vis the self,’ *t*(211) = 1.56, *p* = .121, *d* = .22, 95% CI [−0.10, 0.83], and self-diminishment, *t*(211) = 1.70, *p* = .090, *d* = .23, 95% CI [−0.06, 0.76] (see Table 2).

We next examined our main hypothesis that awe would lead to greater moral expansiveness. First, we found no significant differences in moral expansiveness between participants in the awe condition compared to those in the control condition, *t*(211) = .99, *p* = .322, *d* = .13, 95% CI [−0.16, 0.48]. Following Study 2, we next examined the indirect pathway between conditions (awe vs. control) and moral expansiveness *via* self-reported awe. Following Hayes (2018) bootstrapping procedures, we found a significant indirect pathway, where condition (awe vs. control) promoted greater moral expansiveness through the self-reported experience of awe, *B* = .46, *SE* = .14, 95% CI [0.18, 0.77].

While we did not find a main effect of conditions (awe vs. control) on moral expansiveness, following Study 2, we explored the theoretically predicted indirect pathway through self-reported awe and then the small-self on moral expansiveness. The bootstrapping procedure (Hayes, 2018) revealed there was a significant serial mediation pathway between condition (awe vs. control) and moral expansiveness, through self-reported awe and then the small-self, *B* = .16, *SE* = .08, 95% CI [0.01, 0.31]. Using the same bootstrapping procedure, we next examined the role of the small-self components of ‘vastness vis-à-vis the self’ and ‘self-diminishment.’ We found a significant indirect pathway between condition and moral expansiveness, through self-reported awe and then ‘vastness vis-à-vis the self’ (Figure 1), *B* = .22, *SE* = .08, 95% CI [0.07, 0.40], but not through self-reported awe and then ‘self-diminishment,’ *B* = .03, *SE* = .04, 95% CI [−0.05, 0.13]. This pattern of results was consistent with hierarchical linear regressions, which revealed ‘vastness vis-à-vis the self’ was a significant predictor of moral expansiveness, even while controlling for religiosity and political orientation, whereas ‘self-diminishment’ was a non-significant predictor of moral expansiveness (Table 3).

### Discussion

Similar to Study 2, in Study 3 we found no significant condition (awe vs. control) effect on moral expansiveness. Further, in Study 3 there was also no significant condition (awe vs. control) effect on the small-self. This is inconsistent with prior research which has found similar nature videos induce awe and the small-self (e.g., Piff et al., 2015; Yang et al., 2016). Nonetheless, the presence of a significant indirect pathway through self-reported awe, suggests that insofar as people experienced awe, they also reported a smaller self and greater moral expansiveness, consistent with the findings in Study 2. This suggests that a more powerful awe induction may be required to produce reliable direct effects on the small-self and moral expansiveness.

## Study 4: Virtual reality awe and moral expansiveness

In Study 4, we extended on Studies 2 and 3 by using a more immersive awe induction – a Virtual Reality (VR) simulation of seeing the Earth from near space. The immersive experience of VR has been shown to induce a more intense awe experience compared to 2D video awe inductions (Chirico et al., 2017, 2018). Further, we chose to virtually simulate the ‘overview effect,’ as seeing the Earth from near space has been widely reported by astronauts and cosmonauts to be a profound and transformative awe experience (White, 2014; Yaden et al., 2016).

In Study 4, we also measured trait variables (openness and humility) that may have confounded the causal interpretation of the indirect pathways found in Studies 2 and 3. That is, there may have been trait variables that could have accounted for both higher levels of self-reported awe and moral expansiveness. First, we measured openness (Ashton and Lee, 2009), as openness has been related to both greater dispositional awe (Shiota et al., 2006) and state experiences of awe (Silvia et al., 2015). Further, openness has also been related to greater moral expansiveness (McGrath and Haslam, 2020). Second, we measured humility (Ashton and Lee, 2009), as humility has been linked to both trait and state awe (Stellar et al., 2018). Humility is also likely related to greater moral expansiveness, as humility has been linked to greater moral concern for range of distal entities, such as religious out-group members (Van Tongeren et al., 2016), refugees (Captari et al., 2019), all-humanity and nature (Lee et al., 2015). By controlling for these potentially confounding trait variables, Study 4 could more robustly examine the causal pathway between state experiences of awe and moral expansiveness.

### Method

#### Participants

As we were uncertain about the impact of VR on the main effect size, we followed the power analysis in Studies 1 and 2 and aimed to recruit 200 students. However, as we were only able to recruit until the end of the semester, we only collected data from 126 students. Post-hoc power-analysis, using the medium effect size (*d* = .50) found on moral expansiveness, indicated that the study achieved adequate power (Power = .87) with the recruited sample size (Faul et al., 2009). Participants were students enrolled in a psychology course at an Australian university, who participated in exchange for course credit. One student declined to use the VR and withdrew from the study. The final sample (*N* = 125) comprised 89 women (71.2%) and 36 men (28.8%); ages ranging between 18 and 39 (*M* = 20.06, *SD* = 3.79); 50 identified as East Asian (40%), 33 as White or European (26.4%), 20 as South-east Asian (16%), nine as South Asian or Indian (7.2%), three as Middle Eastern or Arab (2.4%), one as Aboriginal or Torres Strait Islander (0.8%), and one as Latino or Hispanic (0.8%).

#### Materials and procedure

Participants were brought individually into a private room with a computer, which was used by the participant to complete the survey elements of the study. After first giving consent, participants completed a survey containing several trait measures, including their level of openness and humility measured using the 10-item Openness to Experience subscale (e.g., “I like people with unconventional views”; *α* = .74) and the 10-item Honesty-humility (e.g., “Having a lot of money is not especially important to me”; *α* = .72) subscales from HEXACO-60 (Ashton and Lee, 2009). We measured these traits variables as they may have confounded the causal interpretation of the indirect pathways found in Studies 2 and 3.

Participants were then randomly assigned to either a VR-awe condition or non-VR control condition. In the VR-awe condition, participants experienced an immersive virtual simulation of seeing the Earth from near space. This was done using a 360-degree video, from the National Geographic *One Strange Rock* series^10^, which was immersive and allowed viewing each scene from different angles, but notably did not allow interactions with the virtual environment. The 360-degree video was played through an Oculus Rift headset which was connected to a gaming desktop (Intel i7-7700; NVIDIA GeForce GTX 1060 3GB). Additionally, the original audio in the 360-video was muted to remove potentially confounding dialog about global humanism and environmentalism. Instead, participants listened to the Icelandic rock song ‘Hoppípolla’ by Sigur Rós^11^, which has been shown to induce awe-like experiences (Silvia et al., 2015).

Participants in the non-VR control condition were directed to sit at another nearby table to view a model globe for several minutes. The control condition was designed to control for seeing the Earth in the VR-awe condition, which may have been a reminder to some participants about universal or global humanist values. To reduce mind wandering, control participants were given 2-min to count the number of longitude and latitude lines, and then find the most common colors used to denote countries. The use of a non-VR control followed our reasoning that VR itself was a source of awe, as its novel and immersive nature was likely to induce need for accommodation and vastness. Further, the approach was consistent with other recent experiments that have used a VR awe condition and a non-VR control condition (Nelson-Coffey et al., 2019; Kahn and Cargile, 2021). For a detailed outline of the procedures used in the VR awe and non-VR control conditions see Supplementary material.

After completing the experimental condition tasks, participants’ level of moral expansiveness was measured using an adapted version of the Moral Expansiveness Scale (Crimston et al., 2016; *α* = .95). In addition to earlier adjustments made in Study 2, we made further changes to better capture affectively driven changes in moral expansiveness. First, we reasoned that the effects of awe may be diminished through the process of a long introspective survey, so we reduced the number of entities from 30 to 16. The 16 remaining entities remained a face-valid measure of moral expansiveness as they measured moral concern across a range of close human (e.g., citizen of your country), distal human (e.g., refugee), animal (e.g., chimpanzee, bee), plant (e.g., redwood tree), and environmental entities (e.g., coral reef). Second, we removed the category of villains (e.g., child molesters) as these entities were unlike others in having committed a moral crime, and subsequently the relationship between awe and the expansion of moral concern toward these targets is likely to be different than toward relatively innocent entities. The adapted Moral Expansiveness Scale can be found in the Supplementary material.

Participant’s sense of small-self was then measured using the small-self scale (10-item; *α* = .84; Piff et al., 2015) used in Studies 2 and 3, which measured both their small-self sense of ‘vastness vis-à-vis the self’ (5-item; *α* = .86) and ‘self-diminishment’ (5-item; *α* = .76). This was followed by the same emotion manipulation checks used in Studies 2 and 3.

Next, participants completed a final set of demographics which included the same items measuring social conservatism, economic conservatism, and religiosity as Studies 1–3.

### Results

As predicted, participants in the awe condition reported feeling significantly greater awe, compared to participants in the control condition (see Table 2), *t*(123) = 9.84, *p* < .001, *d* = 1.76, 95% CI [2.38, 3.58]. Participants in the awe condition also reported a smaller sense of self compared to those in the control condition (see Table 2), *t*(123) = 4.04, *p* < .001, *d* = .72, 95% CI [0.32, 0.94]. Breaking this down by subscale, this effect was primarily driven by differences in ratings of ‘vastness vis-à-vis the self’ across conditions, *t*(123) = 4.72, *p* < .001, *d* = .84, 95% CI [0.52, 1.27], while there were no significant differences in self-diminishment across conditions, *t*(123) = 1.97, *p* = .051, *d* = .34, 95% CI [−0.00, 0.73].

We next examined our main hypothesis that awe would lead to greater moral expansiveness. We found participants in the awe condition had significantly greater moral expansiveness compared to those in the control condition (Table 2), *t*(123) = 2.79, *p* = .006, *d* = .50, 95% CI [0.17, 1.02]. Further, a hierarchical linear regression model found that condition (awe vs. control) remained a significant predictor of moral expansiveness when controlling for personality (openness, humility) and demographic (political orientations, religiosity) variables (see Table 4).

**Table 4:** Hierarchical linear regression of condition (awe vs. control) and personality in predicting levels of moral expansiveness in Study 4.

Step	Predictor	*B*	*SE*	*β*	*p*	*R* ^2^	*F*	*p*
1						.06	7.48	.007
	Condition^a^	.61	.22	.24	.007			
2						.28	7.39	<.001
	Condition	.51	.20	.21	.012			
	Openness	.51	.18	.24	.005			
	Humility	.70	.18	.31	<.001			
	Religiosity	−.07	.05	−.12	.139			
	Eco. Cons.^b^	−.31	.09	−.31	.001			
	Soc. Cons.^c^	.17	.07	.22	.021			

*Notes*. *N* = 120. B = Unstandardized coefficients. SE = Standard Error of B. β = Standardized coefficients. ^a^ Condition is binary (awe = 1, control = 0). ^b^ Economic conservatism. ^c^ Social conservatism.

**Figure 2:**
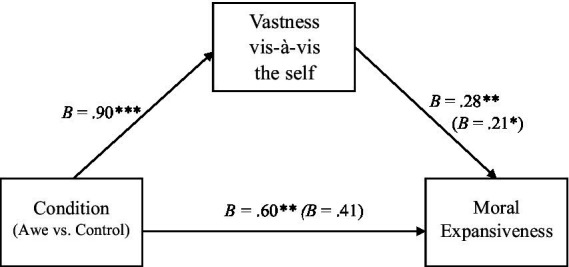
Indirect pathway model through ‘Vastness vis-à-vis the self’ in Study 4. *N* = 125. Predictor variable condition is binary (Awe = 1, Control = 0). Unstandardized coefficients are displayed. Numbers in parentheses indicate coefficients when condition is predicting moral expansiveness while controlling for ‘vastness vis-à-vis the self.’ *p < .05, **p < .01, ***p < .001.

We next examined our secondary hypothesis that awe would promote greater moral expansiveness *via* the small-self. Following Hayes (2018) bootstrapping procedure, we found the small-self was a significant pathway that fully mediated the relationship between condition (awe vs. control) and moral expansiveness, *B* = .16, *SE* = .09, 95% CI [0.01, 0.37]. Breaking this down into the subscale analysis, we conducted two separate indirect pathway analyses using Hayes (2018) bootstrapping procedure. This revealed that ‘vastness vis-à-vis the self’ fully mediated the relationship between condition (awe vs. control) and moral expansiveness (Figure 2), *B* = .18, *SE* = .10, 95% CI [0.01, 0.41]. Self-diminishment was not a significant mediator, *B* = .05, *SE* = .05, 95% CI [−0.03, 0.15].

### Discussion

Study 4 demonstrated that using a more immersive and powerful awe induction (using VR) produced a significant main effect of condition (awe vs. control) on moral expansiveness, even when controlling for personality factors possibly linked with moral expansiveness (openness and humility) as well as demographic variables. By demonstrating that experimentally induced awe increases moral expansiveness, Study 4 provides support for a causal interpretation of the indirect pathways found in Studies 2 and 3. Furthermore, Study 4 demonstrated the relationship between condition (awe vs. control) and moral expansiveness was fully mediated through the small self, and in particular through the small-self sense of ‘vastness vis-à-vis the self,’ again consistent with the indirect effects observed in Studies 2 and 3.

## Cross-study mini meta-analysis: Studies 2–4

**Figure 3:**
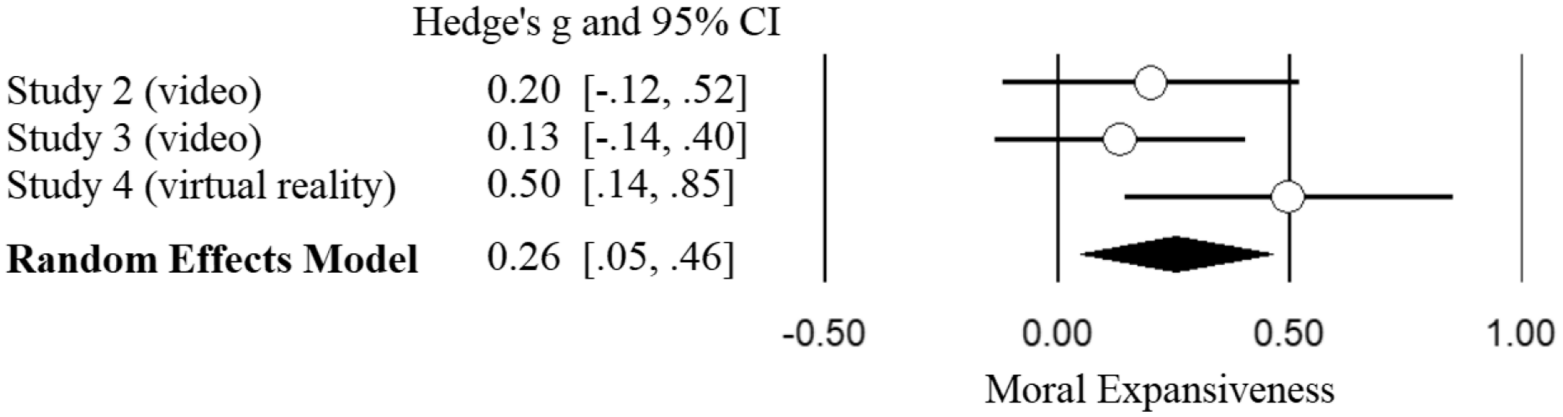
*Notes*. Forest plot of random-effects model of condition (awe vs. control) promoting moral expansiveness in Studies 2–4 (*N* = 505).

To address inconsistencies with regards to the main effects of conditions (awe vs. controls) on moral expansiveness in Studies 2–4, we conducted a mini meta-analysis to examine the overall condition (awe vs. control) effect across Studies 2–4. We chose to conduct a random effects model, to better account for potential differences in condition effects from using either videos (Studies 2 and 3) or VR (Study 4) to induce awe (Riley et al., 2011). We followed the procedures for a mini meta-analysis outlined by Goh et al. (2016) to examine a random effects meta-analysis model across Studies 2–4 (*N* = 505). Overall, the random effects model indicated that those in the awe conditions had significantly greater levels of moral expansiveness, compared to those in the control conditions, Hedge’s *g* = .26, 95% CI [0.05, 0.47], *z* = 2.41, *p* = .016 (see Figure 3 for forest plot). We did not find a significant heterogeneity in effect size, *Q*(2) = 2.67, *p* = .263, *I*^2^ = 25.14%.

Additionally, we examined the overall effects of the small-self across Studies 2–4 using random effects meta-analysis models. These revealed that, compared to those in the control conditions, those in the awe conditions felt a significantly greater sense of small-self overall, Hedge’s *g* = .52, 95% CI [0.22, 0.82], as well as a greater sense of ‘vastness vis-à-vis the self,’ Hedge’s *g* = .54, 95% CI [0.17, 0.91], and self-diminishment, Hedge’s *g* = .33, 95% CI [0.15, 0.50] (for forest plots see Supplementary material).

## General discussion

The experience of awe has been shown to change how people think about themselves and the world around them – feeling a greater sense of connection to a greater whole. We predicted that this rather generalized shift in self-cognition and feelings of connectedness may also have a generalized impact on how people think about their moral worlds, and specifically the expansiveness of those worlds. Across five studies we found support for the role of awe in promoting greater moral expansiveness, and this was in part explained by changes in self-cognition. First, across two correlational studies (Studies 1a and 1b) we found at the trait level, people who experienced greater awe and wonder in their daily lives were also more likely to report greater levels of moral expansiveness. Second, across two experimental studies (Studies 2 and 3), using videos to induce awe, we found consistent indirect pathways, showing that to the extent that people self-reported the experience of awe they also reported greater moral expansiveness, and this was mediated through the small-self sense of ‘vastness vis-à-vis the self’. Finally, in an experimental study using Virtual Reality to induce a more powerful experience of awe (Study 4), we found people in the awe condition (vs. control) reported greater moral expansiveness, and this was fully mediated through the small-self, and again specifically the subscale of ‘vastness vis-à-vis the self.’ A mini meta-analysis across Studies 2–4 revealed evidence of a significant main effect of experimental condition (awe vs. control) on greater moral expansiveness, therefore increasing confidence in the overall pattern of findings.

The present research is the first to demonstrate that awe has a generalized impact on how people structure their moral worlds and the extent of their consideration and concern for a broad range of entities. As predicted, these effects occurred *via* changes in self-cognition, specifically a tendency to experience the self as connected to a greater whole. While past work has shown awe to increase generosity with time and money to specific others (e.g., Piff et al., 2015), or to promote pro-environmental behavioral intentions (e.g., Wang and Lyu, 2019) our findings are the first to demonstrate that awe, and its tendency to change how we think about ourselves, has broader and more generalized implications. That is, we show that awe broadens and deepens people’s moral worlds. Furthermore, in contrast to previous work which found awe promoted pro-sociality (e.g., Piff et al., 2015) and collective engagement (Bai et al., 2017) through the small-self sense of self-diminishment, we found awe promoted greater moral expansiveness through the small-self sense of ‘vastness vis-à-vis the self’. This suggests the pathway through which awe promotes prosocial behavior toward other humans specifically may be different from the pathway through which awe promotes moral concern in a more general sense, including to a range of more distal entities. While awe may promote moral concern to others in our social collective through a sense of humility in relation to something greater (self-diminishment), awe may promote moral concern more broadly, toward all living beings, through a sense of connectedness to a greater whole (vastness vis-à-vis the self).

Our work is also the first to show the impact of emotional states on self-reported moral expansiveness. Consistent with the work of Haidt (2001, 2003) and others, we show that a person’s general moral orientation toward their social and non-social worlds can be impacted by changes in their affective states. This provides a significant extension on prior work which has mostly focused on the effects of disgust or anger on more specific moral judgments (e.g., Rozin et al., 1999; Schnall et al., 2008; Horberg et al., 2011). While previous work has shown the dispositional tendency for empathy (Crimston et al., 2016) or compassion (Crimston et al., 2022) to be related to moral expansiveness, our findings point toward the incidental and situational effects of awe, demonstrating a causal pathway, whereby elevations in the experience of awe promote greater moral expansiveness. As such, our findings provide evidence for an intuitive process through which awe may impact on the size of a person’s moral circle.

The findings extend our understanding of the relationship between the small-self and moral concern toward distal human and non-human entities. Consistent with previous work, we found awe promoted moral expansiveness through the small self. This provided support for our proposed mechanism, and also provides insight into the pathway through which awe may have rather generalized effects on moral cognition.

Taken together, the findings suggest our understanding of awe as a self-transcendent emotion remains incomplete. The prominent social functionalist account suggests that our capacity to experience awe may provide evolutionary advantages by fostering group cohesion and co-ordination (see Stellar et al., 2017). However, this account is hard to square with the finding that awe promotes moral concern toward many distant and non-social entities. Whether this simply represents a spill-over effect, or whether awe serves a function not currently captured within existing accounts is a question for further theorizing and research. Certainly, in this respect, it is noteworthy that the current studies find the effects of awe on moral expansiveness – a generalized shift in the breadth and depth of a person’s moral world – travel through a different component of the small self (i.e., vastness vis-à-vis the self vs. self-diminishment). This difference in process might point to different functions of awe playing out in the case of specific responses to close others vs. more generalized shifts in thinking about a broad range of entities.

One limitation of the present research is that Studies 2 and 3, using videos to induce awe, failed to detect main effects. Instead, we found consistent indirect pathways supporting our main hypotheses, as those who self-reported higher levels of awe also reported greater moral expansiveness. However, in Study 4, using a more powerful VR awe induction, we observed a significant main effect, increasing confidence in the indirect effects observed in Studies 2 and 3. Furthermore, a mini meta-analysis showed a significant effect across all three experimental studies. Our findings attest to the importance of using stronger manipulations when examining the various effects of awe inductions. While videos have been successfully employed in previous research on awe (e.g., Piff et al., 2015), and indeed in a range of studies inducing other types of emotion (e.g., amusement in Valdesolo and Graham, 2014; fear in Gordon et al., 2017), it may be that the broader ranging and more distal effects of awe are more reliably observed using immersive technology (Chirico et al., 2017, 2018).

We also acknowledge the limitation in Study 4, which controlled for content (seeing the Earth as a whole) but did not control for medium (VR awe vs. non-VR control conditions). It is possible the unique features of the VR experience, such as the feeling of presence or immersion (Chirico et al., 2017), may have had an unexpected influence on levels of moral expansiveness. However, such features (e.g., presence) and the general novelty of being immersed in a new virtual space likely promoted the need for accommodation and contributed to higher levels of awe (Chirico et al., 2016), and thereby influence moral expansiveness. While this made the VR itself part of a more intense awe induction, we anticipated difficulties in isolating the effects of awe when using contrasting VR conditions, as any control VR condition was likely to induce some level of awe. Never-the-less, future research should further examine how the unique features of VR can influence moral expansiveness beyond the emotion of awe, and such work may contribute to understanding how VR more generally can be used to promote moral expansiveness.

Experiences of awe are often transformative (Chirico et al., 2022), shifting how we think about ourselves and in turn how we think about our moral worlds. Our findings reveal awe’s capacity to shape in very general ways the breadth and depth of our moral worlds. We find this effect of awe travels through the small-self, and in particular the small-self component of ‘vastness vis-à-vis the self,’ and it is through this broader sense of connectedness to a greater whole that people also feel a greater sense of moral concern for more distant entities, such as out-group humans, animals, plants, and the broader environment.

## Data availability statement

The datasets presented in this study can be found in online repositories. The names of the repository/repositories and accession number(s) can be found at: https://osf.io/hy7ks/.

## Ethics statement

The studies involving human participants were reviewed and approved by The University of Melbourne Human Research Ethics Committee. The participants provided their informed consent to participate in this study. Written informed consent was obtained from the individual(s) for the publication of any identifiable images or data included in this article.

## Author contributions

All authors listed have made a substantial, direct, and intellectual contribution to the work and approved it for publication.

## Conflict of interest

The authors declare that the research was conducted in the absence of any commercial or financial relationships that could be construed as a potential conflict of interest.

## Publisher’s note

All claims expressed in this article are solely those of the authors and do not necessarily represent those of their affiliated organizations, or those of the publisher, the editors and the reviewers. Any product that may be evaluated in this article, or claim that may be made by its manufacturer, is not guaranteed or endorsed by the publisher.

## Footnotes

^1^Exclusions did not affect the significance of zero-order correlations (see Supplementary material).

^2^Exclusions did not affect the significance of zero-order correlations (see Supplementary material).

^3^Significance of the main analyses did not change with the inclusion of the excluded participants (see Supplementary material). Of the 19 exclusions, 15 were in the non-nature awe condition and 4 were from the control condition.

^4^https://vimeo.com/266794879

^5^https://vimeo.com/265573039

^6^https://vimeo.com/265576990

^7^Significance of the main analyses did not change with the inclusion of the excluded participants (see Supplementary material). Nine exclusions were part of the positive awe condition.

^8^https://vimeo.com/266789534

^9^https://vimeo.com/265667914

^10^https://youtu.be/dwHBpykTloY

^11^https://youtu.be/mZTb8WxEW78

## References

[ref1] Adler N. E. Epel E. S. Castellazzo G. Ickovics J. R. (2000). Relationship of subjective and objective social status with psychological and physiological functioning: preliminary data in healthy, White women. Health Psychol. 19, 586–592. doi: 10.1037//0278-6133.19.6.586, PMID: 11129362

[ref2] Ashton M. C. Lee K. (2009). The HEXACO–60: a short measure of the major dimensions of personality. J. Pers. Assess. 91, 340–345. doi: 10.1080/00223890902935878, PMID: 20017063

[ref3] Bai Y. Maruskin L. A. Chen S. Gordon A. M. Stellar J. E. McNeil G. D.. (2017). Awe, the diminished self, and collective engagement: universals and cultural variations in the small self. J. Pers. Soc. Psychol. 113, 185–209. doi: 10.1037/pspa0000087, PMID: 28481617

[ref4] Bastian B. Costello K. Loughnan S. Hodson G. (2012). When closing the human–animal divide expands moral concern: the importance of framing. Soc. Psychol. Personal. Sci. 3, 421–429. doi: 10.1177/1948550611425106

[ref6] Captari L. E. Shannonhouse L. Hook J. N. Aten J. D. Davis E. B. Davis D. E.. (2019). Prejudicial and welcoming attitudes toward Syrian refugees: the roles of cultural humility and moral foundations. J. Psychol. Theol. 47, 123–139. doi: 10.1177/0091647119837013

[ref7] Chirico A. Cipresso P. Yaden D. B. Biassoni F. Riva G. Gaggioli A. (2017). Effectiveness of immersive videos in inducing awe: an experimental study. Sci. Rep. 7, 1–11. doi: 10.1038/s41598-017-01242-0, PMID: 28450730 PMC5430774

[ref8] Chirico A. Ferrise F. Cordella L. Gaggioli A. (2018). Designing awe in virtual reality: an experimental study. Front. Psychol. 8:2351. doi: 10.3389/fpsyg.2017.02351, PMID: 29403409 PMC5786556

[ref9] Chirico A. Pizzolante M. Kitson A. Gianotti E. Riecke B. E. Gaggioli A. (2022). Defining transformative experiences: a conceptual analysis. Front. Psychol. 13:300. doi: 10.3389/fpsyg.2022.790300, PMID: 35814064 PMC9263695

[ref001] Chirico A. Yaden D. B. Riva G. Gaggioli A. (2016). The potential of virtual reality for the investigation of awe. Front. Psychol. 7:1766. doi: 10.3389/fpsyg.2016.01766 27881970 PMC5101419

[ref10] Crimston C. R. Bain P. G. Hornsey M. J. Bastian B. (2016). Moral expansiveness: examining variability in the extension of the moral world. J. Pers. Soc. Psychol. 111, 636–653. doi: 10.1037/pspp0000086, PMID: 26751743

[ref11] Crimston C. R. Blessing S. Gilbert P. Kirby J. N. (2022). Fear leads to suffering: fears of compassion predict restriction of the moral boundary. Br. J. Soc. Psychol. 61, 345–365. doi: 10.1111/bjso.12483, PMID: 34279046

[ref12] Crimston C. R. Hornsey M. J. Bain P. G. Bastian B. (2018a). Toward a psychology of moral expansiveness. Curr. Dir. Psychol. Sci. 27, 14–19. doi: 10.1177/0963721417730888

[ref13] Crimston C. R. Hornsey M. J. Bain P. G. Bastian B. (2018b). Moral expansiveness short form: validity and reliability of the MESx. PLoS One 13:e0205373. doi: 10.1371/journal.pone.0205373, PMID: 30335768 PMC6193647

[ref14] Faul F. Erdfelder E. Buchner A. Lang A. G. (2009). Statistical power analyses using G* Power 3.1: tests for correlation and regression analyses. Behav. Res. Methods 41, 1149–1160. doi: 10.3758/BRM.41.4.1149, PMID: 19897823

[ref16] Goh J. X. Hall J. A. Rosenthal R. (2016). Mini meta-analysis of your own studies: some arguments on why and a primer on how. Soc. Personal. Psychol. Compass 10, 535–549. doi: 10.1111/spc3.12267

[ref002] Goldy S. P. Jones N. M. Piff P. K. (2022). The social effects of an awesome solar eclipse. Psychol. Sci. 33, 1452–1462. doi: 10.1177/09567976221085501 35942889

[ref17] Gordon A. M. Stellar J. E. Anderson C. L. McNeil G. D. Loew D. Keltner D. (2017). The dark side of the sublime: distinguishing a threat-based variant of awe. J. Pers. Soc. Psychol. 113, 310–328. doi: 10.1037/pspp0000120, PMID: 27929301

[ref18] Guan F. Chen J. Chen O. Liu L. Zha Y. (2019). Awe and prosocial tendency. Curr. Psychol. 38, 1033–1041. doi: 10.1007/s12144-019-00244-7

[ref19] Haidt J. (2001). The emotional dog and its rational tail: a social intuitionist approach to moral judgment. Psychol. Rev. 108, 814–834. doi: 10.1037/0033-295X.108.4.814, PMID: 11699120

[ref20] Haidt J. (2003). “The moral emotions” in Handbook of Affective Sciences. eds. Davidson R. J. Scherer K. R. Goldsmith H. H. (Oxford: Oxford University Press), 852–870.

[ref22] Hayes A. F. (2009). Beyond baron and kenny: statistical mediation analysis in the new millennium. Commun. Monogr. 76, 408–420. doi: 10.1080/03637750903310360

[ref23] Hayes A. F. (2018). Introduction to Mediation, Moderation, and Conditional Process Analysis: A Regression-Based Approach (2nd.ed.). New York, NY: The Guilford Press.

[ref24] Horberg E. J. Oveis C. Keltner D. (2011). Emotions as moral amplifiers: an appraisal tendency approach to the influences of distinct emotions upon moral judgment. Emot. Rev. 3, 237–244. doi: 10.1177/1754073911402384

[ref25] Hornsey M. J. Faulkner C. Crimston D. Moreton S. (2018). A microscopic dot on a microscopic dot: self-esteem buffers the negative effects of exposure to the enormity of the universe. J. Exp. Soc. Psychol. 76, 198–207. doi: 10.1016/j.jesp.2018.02.009

[ref27] Kahn A. S. Cargile A. C. (2021). Immersive and interactive awe: evoking awe via presence in virtual reality and online videos to prompt prosocial behavior. Hum. Commun. Res. 47, 387–417. doi: 10.1093/hcr/hqab007

[ref29] Keltner D. Haidt J. (2003). Approaching awe, a moral, spiritual, and aesthetic emotion. Cognit. Emot. 17, 297–314. doi: 10.1080/02699930302297, PMID: 29715721

[ref30] Kirkland K. Crimston C. R. Jetten J. Rudnev M. Acevedo-Triana C. Amiot C. E.. (2022). Moral expansiveness around the world: the role of societal factors across 36 countries. Soc. Psychol. Personal. Sci. doi: 10.1177/19485506221101767

[ref31] Laham S. M. (2009). Expanding the moral circle: inclusion and exclusion mindsets and the circle of moral regard. J. Exp. Soc. Psychol. 45, 250–253. doi: 10.1016/j.jesp.2008.08.012

[ref32] Lee K. Ashton M. C. Choi J. Zachariassen K. (2015). Connectedness to nature and to humanity: their association and personality correlates. Front. Psychol. 6:1003. doi: 10.3389/fpsyg.2015.01003, PMID: 26257669 PMC4508493

[ref33] Luo L. Zou R. Yang D. Yuan J. (2022). Awe experience trigged by fighting against COVID-19 promotes prosociality through increased feeling of connectedness and empathy. J. Posit. Psychol., 1–17. doi: 10.1080/17439760.2022.2131607

[ref34] McGrath M. J. Haslam N. (2020). Development and validation of the harm concept breadth scale: assessing individual differences in harm inflation. PLoS One 15:e0237732. doi: 10.1371/journal.pone.0237732, PMID: 32810186 PMC7437461

[ref35] Neihardt J. G. (2014). Black Elk Speaks. The Complete Edition University of Nebraska Press, Lincoln and London.

[ref36] Nelson-Coffey S. K. Ruberton P. M. Chancellor J. Cornick J. E. Blascovich J. Lyubomirsky S. (2019). The proximal experience of awe. PLoS One 14:e0216780. doi: 10.1371/journal.pone.0216780, PMID: 31121008 PMC6532958

[ref38] Piff P. K. Dietze P. Feinberg M. Stancato D. M. Keltner D. (2015). Awe, the small self, and prosocial behavior. J. Pers. Soc. Psychol. 108, 883–899. doi: 10.1037/pspi0000018, PMID: 25984788

[ref39] Pizarro J. J. Basabe N. Fernández I. Carrera P. Apodaca P. Man Ging C. I.. (2021). Self-transcendent emotions and their social effects: Awe, elevation and Kama Muta promote a human identification and motivations to help others. Front. Psychol. 12:709859. doi: 10.3389/fpsyg.2021.709859, PMID: 34589024 PMC8473748

[ref40] Prade C. Saroglou V. (2016). Awe’s effects on generosity and helping. J. Posit. Psychol. 11, 522–530. doi: 10.1080/17439760.2015.1127992

[ref41] Riley R. D. Higgins J. P. Deeks J. J. (2011). Interpretation of random effects meta-analyses. BMJ 342:d549. doi: 10.1136/bmj.d549 21310794

[ref42] Rohrer J. (2019). Indirect Effect Ex Machina. The 100% CI. Available at: http://www.the100.ci/2019/10/03/indirect-effect-ex-machina/

[ref43] Rozin P. Lowery L. Imada S. Haidt J. (1999). The CAD triad hypothesis: a mapping between three moral emotions (contempt, anger, disgust) and three moral codes (community, autonomy, divinity). J. Pers. Soc. Psychol. 76, 574–586. doi: 10.1037/0022-3514.76.4.574, PMID: 10234846

[ref44] Rucker D. D. Preacher K. J. Tormala Z. L. Petty R. E. (2011). Mediation analysis in social psychology: current practices and new recommendations. Soc. Personal. Psychol. Compass 5, 359–371. doi: 10.1111/j.1751-9004.2011.00355.x

[ref45] Rudd M. Vohs K. D. Aaker J. (2012). Awe expands people’s perception of time, alters decision making, and enhances well-being. Psychol. Sci. 23, 1130–1136. doi: 10.1177/0956797612438731, PMID: 22886132

[ref46] Schnall S. Haidt J. Clore G. L. Jordan A. H. (2008). Disgust as embodied moral judgment. Personal. Soc. Psychol. Bull. 34, 1096–1109. doi: 10.1177/0146167208317771, PMID: 18505801 PMC2562923

[ref47] Shiota M. N. Keltner D. John O. P. (2006). Positive emotion dispositions differentially associated with big five personality and attachment style. J. Posit. Psychol. 1, 61–71. doi: 10.1080/17439760500510833

[ref48] Shiota M. N. Keltner D. Mossman A. (2007). The nature of awe: elicitors, appraisals, and effects on self-concept. Cognit. Emot. 21, 944–963. doi: 10.1080/02699930600923668

[ref50] Silvia P. J. Fayn K. Nusbaum E. C. Beaty R. E. (2015). Openness to experience and awe in response to nature and music: personality and profound aesthetic experiences. Psychol. Aesthet. Creat. Arts 9, 376–384. doi: 10.1037/aca0000028

[ref52] Stellar J. E. Gordon A. Anderson C. L. Piff P. K. McNeil G. D. Keltner D. (2018). Awe and humility. J. Pers. Soc. Psychol. 114, 258–269. doi: 10.1037/pspi0000109, PMID: 28857578

[ref53] Stellar J. E. Gordon A. M. Piff P. K. Cordaro D. Anderson C. L. Bai Y.. (2017). Self-transcendent emotions and their social functions: compassion, gratitude, and awe bind us to others through prosociality. Emot. Rev. 9, 200–207. doi: 10.1177/1754073916684557

[ref54] Valdesolo P. Graham J. (2014). Awe, uncertainty, and agency detection. Psychol. Sci. 25, 170–178. doi: 10.1177/0956797613501884, PMID: 24247728

[ref55] Van Tongeren D. R. Stafford J. Hook J. N. Green J. D. Davis D. E. Johnson K. A. (2016). Humility attenuates negative attitudes and behaviors toward religious out-group members. J. Posit. Psychol. 11, 199–208. doi: 10.1080/17439760.2015.1037861

[ref56] Wang L. Lyu J. (2019). Inspiring awe through tourism and its consequence. Ann. Tour. Res. 77, 106–116. doi: 10.1016/j.annals.2019.05.005

[ref57] White F. (2014). The Overview Effect. Space Exploration and Human Evolution, 3rd. Reston, VA: American Institute of Aeronautics and Astronautics.

[ref58] Yaden D. B. Iwry J. Slack K. J. Eichstaedt J. C. Zhao Y. Vaillant G. E.. (2016). The overview effect: Awe and self-transcendent experience in space flight. Psychol. Conscious. Theory Res. Pract. 3, 1–11. doi: 10.1037/cns0000086

[ref003] Yang Y. Yang Z. Bao T. Liu Y. Passmore H. A. (2016). Elicited awe decreases aggression. J. Pac. Rim Psychol. 10:e11. doi: 10.1017/prp.2016.8

[ref60] Zhao H. Zhang H. Xu Y. Lu J. He W. (2018). Relation between awe and environmentalism: the role of social dominance orientation. Front. Psychol. 9:2367. doi: 10.3389/fpsyg.2018.02367, PMID: 30559692 PMC6286991

[ref004] Zhao H. Zhang H. Xu Y. He W. Lu J. (2019). Why are people high in dispositional awe happier? The roles of meaning in life and materialism. Front. Psychol. 10:1208. doi: 10.3389/fpsyg.2019.01208 31191402 PMC6540826

