# Supplementary material for: Awe promotes moral expansiveness via the small-self
Source: Front Psychol. 2023 Mar 6;14:1097627. doi: 10.3389/fpsyg.2023.1097627 (PMC10025529; doi:10.3389/fpsyg.2023.1097627)
Supplement: Supplementary file 1 [file Data_Sheet_1.pdf]

Supplementary Materials for

Awe promotes moral expansiveness via the small-self

Ji Young Song, Christoph Klebl, Brock Bastian

Correspondence: Ji Young Song, Melbourne School of Psychological Sciences, University of Melbourne, Victoria, 3010, Australia, songjy@student.unimelb.edu.au , or being.jisong@gmail.com

Table of Contents

Open Access Data OSF links and notes (p.3)

Study 1a: Dispositional Awe and Moral Expansiveness (USA)

Analysis without exclusions (p.3)

Correlations b/w Moral Exp, Disp. Awe, and Demographics (p.3)

Regression Disp. Awe & Demographics Predicting Moral Exp. (.4)

Study 1b: Dispositional Awe and Moral Expansiveness (Australia)

Analysis without exclusions (p.5)

Correlations b/w Moral Exp, Disp. Awe, and Demographics (p.5)

Intercorrelations b/w Moral Exp.[1] & Disp. for Positive Emotions (p.6)

Regression Disp. Awe, Demo., & Other Pos. Emo. Predicting Moral Exp. (p.7)

Study 2: The experience of awe and moral expansiveness (videos, Australia)

Analysis without exclusions (p.8)

Modified Moral Expansiveness Scale (p.10)

Mean Self-reported Emotional States in Studies 2, 3, & 4 (p.13)

Study 3: Nature induced awe and moral expansiveness (videos, USA)

Analysis without exclusions (p.14)

Study 4: Virtual Reality Awe and Moral Expansiveness (VR, Australia)

Analysis without exclusions (p.15)

Adapted Moral Expansiveness Scale (p.16)

Intercorrelations b/w Awe, Moral Expansiveness, & Small-self (p.17)

Self-reported awe mediates conditions and moral exp. (p.17)

Main effect across entities

VR Training Procedures of Research Assistants (p.21)

Procedures for VR awe and non-VR control conditions (p.23)

Images from study (p.27)

Mini meta-analyses small-self, vastness, self-diminishment Studies 2-4 (p.28)

Attention Checks used across Studies 1-4 (p.29)

Supplementary References (p.30)

[1] Moral Expansiveness

OSF files

Open access to the finalized sample data, SPSS syntax, and Qualtrics surveys used can be found at the following Open Science Framework (OSF) link: https://osf.io/hy7ks/

Study 1a: Dispositional Awe and Moral Expansiveness (USA)

Analysis without exclusions

We conducted zero-order correlations, without exclusions, and found there was a significant positive relationship between disposition for awe and moral expansiveness (Table 1). Further, partial correlations found the relationship between disposition awe and moral expansiveness remained significant while controlling for religiosity, economic conservatism, and social conservatism, $r(197) = .27$, $p < .001$.

Supplementary Table 1

Zero Order Correlations between Disposition for Awe, Moral Expansiveness, Religiosity, Economic Conservatism, and Social Conservatism in Study 1a (USA)

Variable	1.	2.	3.	4.	5.	6.
1. Disp. Awe[a]	-					
2. Moral Exp.[b]	.28***	-				
3. Religiosity	.23**	.02	-			
4. Eco. Conserv.[c]	.14*	-.15*	.55***	-		
5. Soc. Conserv.[d]	.20*	-.11	.59***	.87***	-	
6. Status[e]	.30***	.07	.40***	.41***	.40***	-
Mean	4.92	1.67	3.87	3.97	3.75	5.57
SD	1.14	0.42	2.73	2.01	2.10	2.44

Note. $N = 202 – 208$. a. Disposition for Awe. b. Moral Expansiveness. c. Economic conservatism d. Social conservatism. e. Self-perceived social status. *** $p < .001$. ** $p < .01$. * $p < .05$.

Supplementary Table 2

Hierarchical Linear Regressions of Dispositional Awe and Demographic Variables Predicting Levels of Moral Expansiveness in Study 1a (USA)

Variable	*B*	*SE*	β	*t*	*p*-value
Model 1					
(Constant)	1.28	.13		9.59	.000
Dispositional Awe	.11	.03	.30	4.25	.000
Conservatism	-.04	.02	-.19	-2.45	.015
Religiosity	.012	.01	.07	1.01	.312
Social Status	-.01	.02	-.04	-0.51	.614
Model 2					
(Constant)	1.05	.17		6.29	.000
Dispositional Awe	.11	.03	.30	4.26	.000
Conservatism	-.04	.02	-.21	-2.67	.008
Religiosity	.01	.01	.06	0.80	.424
Social Status	-.01	.02	-.02	-0.30	.762
Age	.00	.00	.10	1.32	.187
Gender	.09	.05	.14	2.01	.046
Ethnicity	-.01	.02	-.04	-.53	.596

Note. Regression conducted with exclusions, N = 193.

Study 1b: Dispositional Awe and Moral Expansiveness (Australia)

Analysis without exclusions

We conducted zero-order correlations, without exclusions, and found there was a significant positive relationship between disposition for awe and moral expansiveness (Table 3).

Supplementary Table 3

Zero Order Correlations between Disposition for Awe, Moral Expansiveness, Religiosity, Economic Conservatism, and Social Conservatism in Study 1b (Without Exclusions)

Variable	1.	2.	3.	4.	5.	6.
1. Disp. Awe[a]	-					
2. Moral Exp.[b]	.12*	-				
3. Religiosity	.05	.01	-			
4. Eco. Conserv.[c]	-.03	-.16**	.14**	-		
5. Soc. Conserv.[d]	-.04	-.15**	.24***	.58***	-	
6. Status[e]	.16**	.00	-.04	-.03	-.09	-
Mean	4.77	1.74	2.39	3.28	2.72	6.71
SD	1.01	0.46	2.19	1.35	1.58	2.04

Note. N = 331-352. a. Disposition for Awe. b. Moral Expansiveness. c. Economic conservatism d. Social conservatism. e. Self-perceived social status. *** $p < .001$. ** $p < .01$. * $p < .05$.

Intercorrelations between Moral Expansiveness and Disposition for Positive Emotions

Supplementary Table 4

Intercorrelations between Moral Expansiveness and Disposition for Positive Emotions in Study 1b (Australia)

Variable	1.	2.	3.	4.	5.	6.	7.	8.
1. Moral Exp.	-							
2. Disp. Awe	.17**	-						
3. Disp. Joy	.10	.50***	-					
4. Disp. Contentment	.00	.42***	.72***	-				
5. Disp. Pride	.03	.40***	.57***	.67***	-			
6. Disp. Love	.03	.32***	.60***	.48***	.51***	-		
7. Disp. Compassion	.23***	.33***	.40***	.23***	.34***	.44***	-	
8. Disp. Amusement	.11	.13*	.33***	.15*	.18**	.31***	.36***	-
Mean	1.76	4.78	4.42	4.32	4.68	4.59	5.68	4.90
SD	0.44	1.02	1.06	1.16	1.00	1.14	0.86	1.09

Note. N = 283-288; Disp. = Disposition; *** $p < .001$ ** $p < .01$ * $p < .05$.

Supplementary Table 5

Hierarchical Linear Regressions of Dispositional Awe, Demographic Variables, and Disposition for Other Positive Emotions Predicting Levels of Moral Expansiveness in Study 1b (Australia)

Variable	*B*	*SE*	β	*t*	*p*-value
Model 1					
(Constant)	1.65	.20		8.20	< .000
Dispositional Awe	.07	.03	.16	2.27	.024
Conservatism	-.07	.02	-.20	-3.13	.002
Religiosity	.02	.01	.10	1.58	.117
Social Status	-.00	.01	-.01	-.12	.902
Dispositional Joy	.04	.04	.10	.95	.343
Disp. Contentment	-.03	.04	-.09	-.88	.381
Dispositional Pride	-.02	.04	-.03	-.39	.697
Dispositional Love	-.03	.03	-.08	-1.01	.312
Disp. Amusement	.03	.03	.06	.92	.356
Model 2					
(Constant)	1.35	.23		5.98	< .001
Dispositional Awe	.05	.03	.12	1.75	.081
Conservatism	-.06	.02	-.18	-2.85	.005
Religiosity	.02	.01	.08	1.36	.174
Social Status	-.00	.01	-.01	-.08	.934
Dispositional Joy	.03	.04	.07	.70	.483
Disp. Contentment	-.02	.04	-.05	-.55	.584
Dispositional Pride	-.03	.04	-.07	-.77	.444
Dispositional Love	-.05	.03	-.13	-1.64	.102
Disp. Amusement	.01	.03	.02	.31	.760
Disp. Compassion	.11	.04	.21	2.86	.005

Note. Regression conducted with exclusions, $N = 263$.

Study 2: The experience of awe and moral expansiveness

Analyses without exclusions

Initial zero-order correlations indicated that overall there was a significant positive relationship between self-reported awe and moral expansiveness, $r(180) = .18$, $p = .016$, and also between self-reported awe and small-self, $r(183) = .30$, $p < .001$. Examining the components of the small-self, we found that there was a significant relationship between self-reported and both the small-self components of 'vastness vis-à-vis the self', $r(183) = .28$, $p < .001$, and self-diminishment, $r(184) = .21$, $p = .004$.

Next, we examined condition differences in levels of self-reported awe, moral-expansiveness, and small-self. First, we found there were condition differences in levels of self-reported awe, $F(2, 182) = 36.14$, $p < .001$. Second, we found there were no condition differences in levels of moral expansiveness, $F(2, 180) = 1.05$, $p = .353$. Third, we found there were condition differences in levels of small-self, $F(2, 183) = 14.75$, $p < .001$. Examining the components of the small-self, we found there were condition differences in both levels of 'vastness vis-à-vis the self', $F(2,183) = 10.64$, $p < .001$, and self-diminishment, $F(2, 183) = 9.96$, $p < .001$.

Using a dummy coded condition variable (0 = control, 1 = awe), we next conducted bootstrapping procedures to examine indirect pathways between conditions (awe vs. control) and moral expansiveness via self-report awe and then then small-self. First, the bootstrapping procedures revealed that self-report awe was a significant indirect pathway between conditions (awe vs. control) and moral expansiveness, $B = .18$, $SE = .09$, 95% CI [.00, 38]. Next examining the serial mediation pathways, bootstrapping procedures revealed that there was a significant indirect pathway between conditions (awe vs. control) and moral

expansiveness, first through self-report awe and then through the small-self, *B* = .04, *SE* = .03, 95% CI [.00, .10].

Examining the serial mediation pathways through the small-self components, we first found there was a significant indirect pathway between conditions (awe vs. control) and moral expansiveness, through self-report awe and then ‘vastness vis-à-vis the self’, *B* = .05, *SE* = .03, 95% CI [.01, .12], however the indirect pathway through self-report awe and self-diminishment was not significant, *B* = 00, *SE* = .02, 95% CI [-.03, .05].

Modified Moral Expansiveness Scale (modified version of Crimston et al., 2016)

How much **moral concern** do you ***feel*** for the following entities?

Extreme moral concern means that you have ***a great desire to protect them*** and have a ***high-level of care*** about their ***welfare***.

No moral concern means that you have ***little desire to protect them*** and al ***low-level of care*** about their ***welfare.***

No moral concern						Extreme Moral Concern
1	2	3	4	5	6	7

m-MES Entity List (United States)	m-MES Entity List (Australian)
Family/Friends	Family/Friends
Family member	Family member
Close friend	Close friend
Partner/spouse	Partner/spouse
In-group	In-group
American citizen	Australian citizen
Somebody from your neighbourhood	Somebody from your neighbourhood
Co-worker	Co-worker
Outgroup	Outgroup
Foreign citizen	Foreign citizen
Member of opposing political party	Member of opposing political party
Somebody with different religious beliefs	Somebody with different religious beliefs

(continued)

m-MES Entity List (United States)	m-MES Entity List (Australian)
Revered	Revered
U.S. President (position not specific individual)	Prime Minister of Australia (position not specific individual)
U.S. soldier	Australian soldier
Charity worker	Charity worker
Stigmatized	Stigmatized
LGBT+ individual	LGBT+ individual
Intellectually impaired person	Intellectually impaired person
Refugee	Refugee
Villains	Villains
Murderer	Murderer
Terrorist	Terrorist
Child molester	Child molester
Animals high-sentient	Animals high-sentient
Chimpanzee	Chimpanzee
Dolphin	Dolphin
Cow	Cow
Animals low-sentient	Animals low-sentient
Chicken	Chicken
Fish	Fish
Bee	Bee

(continued)

m-MES Entity List (United States)	m-MES Entity List (Australian)
Plants	Plants
Redwood tree	Redwood tree
Apple tree	Apple tree
Rose bush	Rose bush
Environment	Environment
Coral reef	Coral reef
Old-growth forest	Old-growth forest
Grand Canyon National Park	Uluru (Ayers Rock)

Supplementary Table 6

Mean Scores for Self-reported Emotional States in Studies 2, 3, and 4 (Standard Deviations in Parentheses)

	Study 2 Videos			Study 3 Videos		Study 4 Virtual Reality	
	Neg. Nat. Awe (*n* = 63)	Non-nat. Awe (*n* = 47)	Control (*n* = 57)	Pos. Nat. Awe (*n* = 93)	Control (*n* = 120)	VR Awe (*n* = 62)	Control (*n* = 63)
Amusement	2.81 (2.04)	3.89 (1.87)[ac]	2.77 (1.78)	2.96 (1.89)	2.58 (1.73)	5.03 (1.56)[c]	3.30 (1.79)
Anger	2.18 (1.45)[bc]	1.21 (0.62)	1.35 (1.03)	1.19 (0.66)	1.31 (1.07)	1.13 (0.59)	1.24 (0.84)
Anxiety	3.82 (1.76)[bc]	1.74 (1.22)	2.07 (1.74)	1.32 (0.96)	1.54 (1.35)	2.35 (1.63)	2.52 (1.77)
Awe	5.02 (1.73)[bc]	4.72 (2.11)[c]	2.40 (1.70)	5.20 (1.89)[c]	2.58 (1.71)	5.58 (1.72)[c]	2.60 (1.66)
Boredom	-	-	-	-	-	1.56 (0.90)	2.70 (1.67)[d]
Disgust	1.98 (1.35)[c]	1.57 (1.18)	1.42 (1.03)	1.18 (0.78)	1.31 (1.06)	1.18 (0.69)	1.13 (0.49)
Fear	4.34 (1.85)[bc]	1.53 (1.00)	1.88 (1.57)	1.31 (0.88)	1.34 (0.99)	2.18 (1.55)	1.78 (1.39)
Nervousness	4.13 (1.71)[bc]	1.91 (1.37)	2.44 (1.73)	1.26 (0.87)	1.44 (1.21)	3.05 (1.82)	2.94 (1.79)
Pride	2.15 (1.68)[c]	2.19 (1.59)[c]	1.39 (0.82)	3.27 (2.08)[c]	2.33 (1.70)	3.81 (1.85)[c]	2.22 (1.58)
Sadness	3.89 (1.99)[bc]	1.64 (1.11)	1.33 (1.02)	1.59 (1.31)	1.41 (1.19)	1.95 (1.27)	1.60 (1.07)
Happiness	2.44 (1.59)	4.32 (1.59)[ac]	2.11 (1.41)	4.86 (1.77)[c]	3.43 (1.92)	5.45 (1.30)[c]	3.03 (1.65)

Notes. a. These means are significantly greater than those in negative nature awe, $ps < .05$.
b. These means are significantly greater than those in the non-nature awe condition, $ps < .05$.
c. These means are significantly greater than those in the control condition, $ps < .05$.
d. These means are significantly greater than those in the awe condition, $ps < .05$

Study 3: Nature induced awe and moral expansiveness

Analyses without exclusions

First we examine the zero-order correlations between self-report awe, moral expansiveness, and the small-self. We found self-report awe was significantly positively related to both levels of moral expansiveness, *r*(220) = .26, *p* < .001, and small-self, *r*(220) = .29, *p* < .001. Examining the relationship between self-report and the subscales of the small-self, we found self-report awe was significantly positively related to levels of 'vastness vis-à-vis the self', *r*(220) = .33, *p* < .001, and self-diminishment, *r*(220) = .16, *p* < .001.

We examined whether there were differences between conditions in levels of moral expansiveness and small-self. First, we found there was no significant difference in levels of moral expansiveness between participants in the awe condition (*M* = 4.49, *SD* = 1.27, *n* = 102) compared to the control condition (*M* = 4.39, *SD* = 1.12; *n* = 120), *t*(220) = 0.64, *p* = .526. Second, we found participants in the awe condition (*M* = 2.49, *SD* = 1.41) had a significantly stronger sense of small-self, compared to those in the control condition (*M* = 4.12, *SD* = 1.32), *t*(220) = 2.03, *p* = .044. However, at the subscale levels, we found there was no significant difference in 'vastness vis-à-vis the self' between the awe condition (*M* = 4.58, *SD* = 1.64) and control conditions (*M* = 4.17, *SD* = 1.73), *t*(220) = 1.80, *p* = .073, and we found there was no significant difference in 'self-diminishment' between the awe condition (*M* = 4.39, *SD* = 1.55) and control conditions (*M* = 4.06, *SD* = 1.43), *t*(220) = 1.66, *p* = .098.

While there was no direct main effect, the pattern of correlations were consistent with an indirect pathway model. Further, Hayes (2009) has demonstrated the presence of indirect pathways in the absence of main effects. Therefore, to examine our main hypotheses further, we explored the mediation pathways using bootstrapping procedures (Hayes, 2018). First, we found self-report awe was a significant indirect pathway between conditions (awe vs. control)

and moral expansiveness, *B* = .49, *SE* = .22, 95% [.22, .78]. Examining the serial mediation pathway, we found there was a significant indirect pathway between conditions (awe vs. control) and moral expansiveness, first through self-report awe and then the small-self, *B* = .16, *SE* = .08, 95% CI [.02, .32].

We next explored the serial mediation pathways at the sub-scale levels of the small-self. First, we found that there was a significant indirect pathway between conditions (awe vs. control) and moral expansiveness, first through self-report awe and then the small-self sense of 'vastness vis-à-vis the self', *B* = .22, *SE* = .08, 95% CI [.07, 39]. However, we found the indirect pathway between conditions and moral expansiveness, through self-report awe and then self-diminishment, was not significant, *B* = .04, *SE* = .04, 95% CI [-.04, .13].

While no main effect on moral expansiveness was found between conditions (awe vs. control), the significant indirect pathways suggest that insofar as people felt greater levels of awe, they also felt a stronger sense of small-self – and in particular the small-self sense of 'vastness vis-à-vis the self' – which in turn predicted greater levels of moral expansiveness.

Study 4: Virtual Reality Awe and Moral Expansiveness (VR, Australia)

Analysis without exclusions

In Study 4, only 1 participant was excluded because they declined to use the VR and withdrew their participation in the remainder of the study. Subsequently, they did not provide responses to the moral expansiveness or small-self scales to conduct a comparison without exclusions analysis. There were no attention checks in the Study 4 survey.

Adapted Moral Expansiveness Scale (adapted from Crimston et al., 2015)

Right now, how do you ***feel like caring for*** a:

Not at all	-	-	-	-	-	Extremely
1	2	3	4	5	6	7

1. Citizen of your country
2. Co-worker
3. Member of opposing political party
4. Person with different religious belief
5. Citizen of a foreign country
6. LGBT+ person
7. Intellectually impaired person
8. Refugee
9. Chimpanzee
10. Cow
11. Fish
12. Bee
13. Redwood tree
14. Apple tree
15. Coral reef
16. Old-growth forest

Supplementary Table 7

Intercorrelations between Self-report Awe, Moral Expansiveness, the Small-self

Variable	1	2	3	4	5
1. Awe[a]	-				
2. Moral Expansiveness	.36***	-			
3. Small-self	.30**	.25**	-		
4. Vastness[b]	.32***	.26**	.86***	-	
5. Self-diminishment	.16[†]	.16[†]	.82***	.41***	-

Note. N = 125; a = Self-report awe; b = Vastness vis-à-vis the self; *** p < .001. ** p < .01. * p < .05. † p < .10

Self-reported awe mediates pathway between conditions (awe vs. control) and moral expansiveness

We examined whether self-reported awe specifically mediated the pathway between condition (awe vs. control) and moral expansiveness. We followed the Hayes (2018) bootstrapping procedure using Model 4 in the SPSS PROCESS macro. Self-reported awe fully mediated the pathway between condition (awe vs. control) and moral expansiveness (Figure 1), B = .58, SE = .19, 95% CI [.24, .98]. The analysis supported the interpretation that it was the difference in self-reported awe between conditions driving effects on moral expansiveness.

Supplementary Figure 1

Mediation Model through Self-report Awe in Study 4

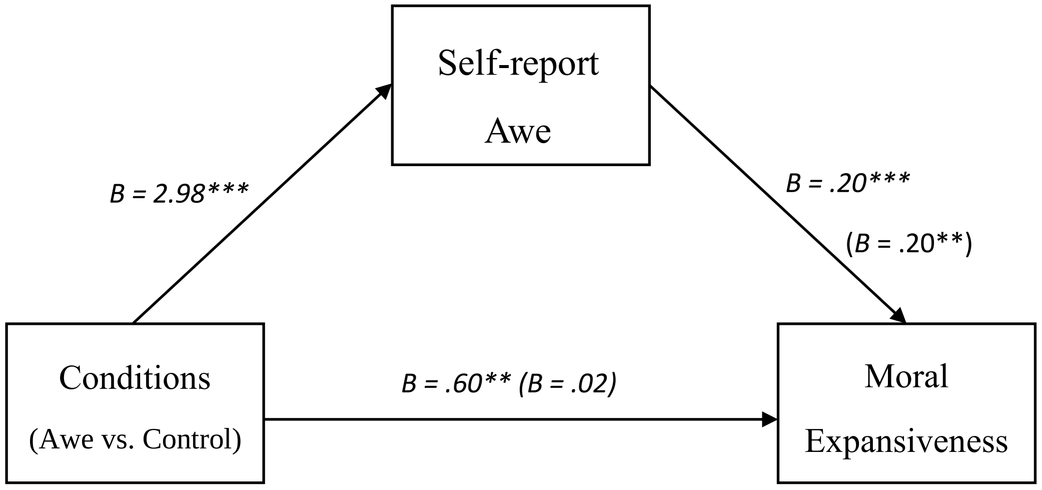

Notes. N = 125. Predictor variable condition is binary (Awe = 1, Control = 0). Unstandardized coefficients are displayed. Numbers in parentheses indicate coefficients when condition is predicting moral expansiveness while controlling for self-report awe. * $p < .05$. ** $p < .01$. *** $p < .001$

Main effects across entities

To examine whether awe was broadly increasing moral concern across a range of entity types, or whether it was specific to some entities, we explored the main effects (awe vs. control conditions) on human and non-human entities separately, as well with each separate entity type. First, we found there was only a marginally significant difference in moral concern towards the subset of eight human entity types (family, co-worker, member of opposing political party, person with different religious beliefs, citizen of a foreign country, intellectually impaired person, refugee) between the awe (*n* = 62; *M* = 5.11, *SD* = 1.18) and control conditions (*n* = 63; *M* = 4.67, *SD* = 1.39), *t*(123) = 1.91, *p* = .059, *d* = .34. Next, examining the subset of non-human entities (chimpanzee, cow, fish, bee, redwood tree, apple

tree, coral reef, and old-growth forest) we found there was a significant difference in moral concern towards the non-human entities between the awe (n = 60; M = 4.67, SD = 1.20) and control conditions (n = 60; M = 4.02, SD = 1.45), t(3.17) = 118, p = .002, d = .49.

Examining main effects on each entity type separately (Supplementary Table 8) we found among the human entities there were significant differences in moral concern towards people with different religious beliefs, foreign citizens, and intellectually impaired persons. Amongst non-human entities, we found significant differences in moral concern for fish, bee, cow, redwood tree, apple tree, coral reef, and old-growth forest. That is, we found significant differences across more non-human entity types than human entity types. It may be that moral concern towards distal non-human entities are more flexible to changes in moral concern compared to relatively fixed beliefs people nay have towards other human entities.

Supplementary Table 8

Means and Standard Deviations of Moral Concern for Human and Non-human Entities in Study 4

	Control	Awe	
Entity	*M (SD)*	*M (SD)*	*p*
Citizen of your country	5.13 (1.45)	5.31 (1.31)	.470
Co-worker	5.08 (1.53)	5.47 (1.26)	.124
Member political opposition	3.54 (1.75)	4.06 (1.64)	.086
Person different religion	4.52 (1.65)	5.08 (1.45)	.048
Foreign citizen	4.54 (1.58)	5.10 (1.48)	.044
LGBT+ person	4.89 (1.77)	5.32 (1.49)	.141
Intellectually impaired person	4.83 (1.78)	5.47 (1.38)	.026
Refugee	4.84 (1.86)	5.08 (1.68)	.452
Chimpanzee	4.43 (1.90)	4.81 (1.50)	.220
Cow	3.84 (1.70)	4.32 (1.53)	.099
Fish	3.75 (1.73)	4.67 (1.64)	.003
Bee	3.50 (1.92)	4.21 (1.72)	.032
Redwood tree	3.66 (1.89)	4.68 (1.61)	.002
Apple tree	3.62 (1.78)	4.60 (1.56)	.001
Coral reef	4.63 (1.86)	5.34 (1.53)	.022
Old-growth forest	4.61 (1.70)	5.52 (1.40)	.002

Procedures for training Research Assistants and interns

Research Assistants (RAs) and interns were used to run participants through the lab experiment. To get them familiar with the VR awe and non-VR awe conditions, there were several steps to their training. First, prior to starting their training, RAs were emailed the 'Oculus Rift Safety & Warranty Manual' and "VR Experiment Procedures" (p.19) and asked to become familiar with both documents.

RAs were then scheduled for a one-on-one training session with one of the main researchers who was familiar with VR. RAs were first shown where participants would be standing in the lab (denoted by an X marked by masking tape on the floor), and shown how to ensure that the room was clear of trip hazards. RAs were then handed the Oculus Rift headset and instructed in how to adjust the headset so that it was secure and the vision was clear. An emphasis was placed on becoming familiar with the three adjustment points; adjusting the inter-pupillary distance (IPD) using the slider at the bottom of the headset, adjusting the strap at the top of the headset to adjust the height of the headset on the user face, and using the straps on the sides to secure the headset firmly. RAs were then handed the controllers and instructed how to navigate to the Samsun VR video player and shown how to play several 360 videos. This included how to open a video, set it to mute, return the video to the start, and return to the main menu of the Samsung VR player. This was to ensure that the RA would know how to set up the VR condition prior to each participant, so that each participant would only have to click on a video icon to start the 360 video and have it play without sound. RAs were then shown how to setup Spotify and ensure the music was being piped into the Oculus Rift headset. They were then shown how to have Hoppipolla ready on hand, so that when the participant played the VR video, they could pipe in Hoppipolla from Spotify at the same time. RAs were then shown the non-VR condition and shown how to run participants through the control condition. Following this, the RA was asked to practice

running both the VR awe and control conditions using the instructor as a participant. This was done several times, with feedback given to the RA on each practice run. An emphasis was placed on RAs being confident, polite, and neutral in both conditions. Finally, RAs were shown the cleaning procedures of the Oculus Rift headset and how to set up both conditions prior to each participant, with an emphasis placed on setting up the headset so that it was already in the Samsung Video player and Spotify ready to play Hoppipolla.

To continue their training, each RA then first shadowed one of the main researchers through at least 3 participants, where they silently observed one of the main researchers conduct the experiment. This was followed by the RA then running at least 3 participants with one of the main researchers shadowing them. Participants were all asked if they consented to an RA observing the experiment (when the RA was conducting the experiment, the main researcher was made to be the RA shadowing a researcher). After each of these sessions a debrief session was conducted between the RA and one of the main researchers. An emphasis was again placed on the RA being confident, polite, and neutral in both conditions.

Procedures for VR awe and non-VR control conditions

Below is the procedural script given to the interns and research assistants who were running participants through Study 4. After initially completing some personality surveys, participants were randomly assigned either a large black circle or grey circle. These circles both contained instructions to call the researcher in to continue the experiment. If the researcher saw a black circle, they proceeded to run the participant through the VR awe condition. If the interns saw a grey circle, they proceeded to run the participant through the control condition.

Note: Prior to each participant, the interns and research assistants set-up the *Spotify* music player to play '*Hoppípolla*' by Sigur Rós[2] with one click. Next, they set up the *Samsung VR* player so that the *One Strange Rock*[3] video was muted and ready to be played from the menu by one click.

VR Awe Experiment Procedure

[When the participant arrives at the lab, first ensure that they are the correct person booked into the session. Then bring the participant into the experiment, while follow the Initial script. After completing the Initial script, close the door and wait outside for the participant to get you.]

Initial

"It's all set. Read the plain language statement and then the consent form. After that it will take you straight through to some questionnaires. There is plenty of time, so read all the questions carefully and answer them as honestly as possible.

---

[2] https://youtu.be/mZTb8WxEW78

[3] https://youtu.be/dwHBpykTloY

Half-way through, there will be a prompt to call in the researcher. I'll just be sitting outside. Thanks"

[When the participant calls you into the room, first check whether the circle is grey or black on the screen. Follow the appropriate Conditions script accordingly.]

Conditions

Grey = Control condition

"For the next part of the experiment, we would like you to sit at this table. [*wait for participant to sit in the chair. Take a seat in the other nearby chair*]. I am going to give you a task. In 2 minutes, I want you to study the globe in front of you. First, count the number of longitude and latitude lines. Next, I want you work out which colour is the most commonly used for countries, which colour is 2nd most common for countries, and which colour is 3rd most for countries. You don't need to give me your answers, we will ask you at the end of the survey. The shade of colour doesn't matter. And size of the country doesn't matter either. Okay start.

[*Give count-down warning at 1 min*] "You have 1 minute left".

[*Give count-down warning at 30 seconds*] "You have 30 seconds left".

"You can return to the computer. Click next, and it will take you through to the rest of the experiment. Please answer the questions as honestly as possible."

Black = Awe condition

"As part of the experiment, we want to know how people experience virtual reality. Would it be okay to put you through a virtual reality experience?"

[**Note:** *If participants decline, then end the experiment. Thank the student for their participation and let them know that they will still be receiving the REP credit for their participation*.]

“Please stand on the white tape X on the floor.”

[*Indicate the white X marked on the floor and wait for the participant to stand on the X*.]

“So we are going to put you through a virtual reality video. It is not the kind of VR experience in which you will need to walk around, but it is a 360 experience. So, there will be things all around you to see. So, feel free to turn around, if you feel like it. [*Move 360 in front participant*]. Move your head up and down. Really feel free to look all around. [*Move head up and down in front of participant*]. There may be times where there is a better view behind you, so make sure to all around sometimes!

[*Pick up and show the participant the right-hand controller*]. It’s also not a very interactive video, however, you will need this controller to get the video started in the beginning. All you need to know is that its like a pointer, and you just use the trigger button at the front [*show participant the trigger button*] to click on things. At the start, I will place the controller in your right hand and ask you to start to the video. After that I will take the controller out of your hands.

[*Show the participant the Oculus Rift headset*]. This an Oculus Rift headset. There are 3 main parts to adjusting it. First, there is a switch underneath the right eye that you can move left and right that controls how far apart the lens are. I find that it is best to start adjusting this first. There should be a setting which makes things clearer. Next, start to move the Oculus up and down your face until you find the position that makes things the most clear. Then you can use the strap at the top to lock in the height. Next, you can use the straps on the side to tighten the Oculus around your head. It should sit comfortably and feel secure without you needing

to hold the headset with your hands. [*Pass the headset to participant*]. Okay. You can start adjusting, we have plenty of time, so take your time to get it clear and comfortable.

[*When participant is ready, pass them the controller*]. Okay can you see the video that starts "First 3D VR…". Could you point and click on that video. [*Play Hoppipolla via Spotify*.]

[*Watch the progress of the VR through the viewer program. When they are done, stop playing Hoppipolla on Spotify*.]

You can return to the computer. Click next, and it will take you through to the rest of the experiment. Please answer the questions as honestly as possible."

[*Leave the experiment room*.]

Images from Study 4

Supplementary Figure 2

Images of the VR awe condition. Left panel shows a participant using the Oculus Rift. Right panel shows an example of the footage seen in the VR simulation.

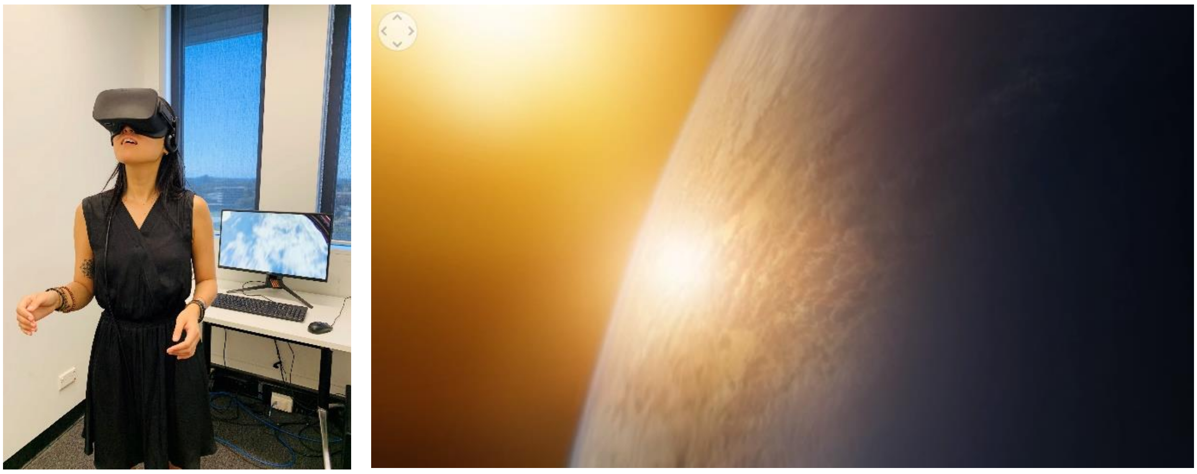

Supplementary Figure 3

Model globe used in the non-VR control condition

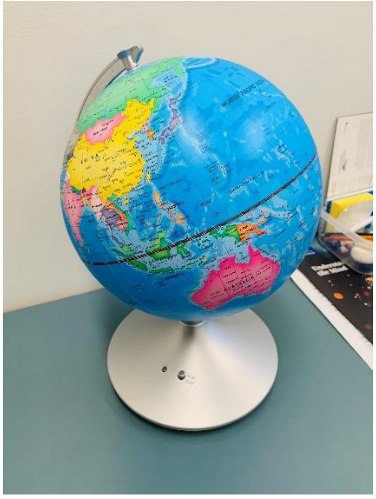

Mini meta-analysis for small-self and its components

Mini meta-analyses conducted using Comprehensive Meta Analysis software Version 3.3.070.

Supplementary Figure 4

Forest Plot of Random-effects Model of Condition (Awe vs. Control) Promoting Small-self in Studies 2-4 (N = 505)

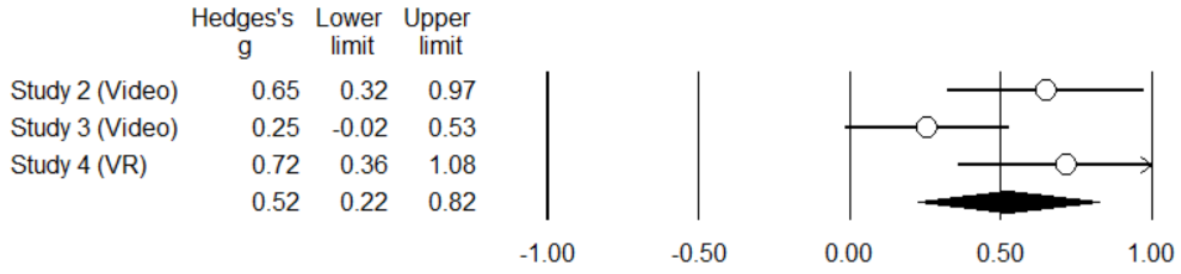

Supplementary Figure 5

Forest Plot of Random-effects Model of Condition (Awe vs. Control) Promoting Small-self component ‘Vastness vis-à-vis the self’ in Studies 2-4 (N = 505)

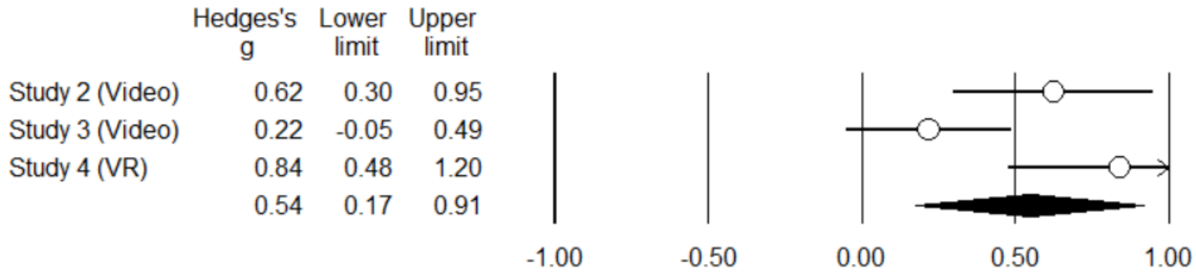

Supplementary Figure 6

Forest Plot of Random-effects Model of Condition (Awe vs. Control) Promoting Small-self component ‘Self-diminishment’ in Studies 2-4 (N = 505)

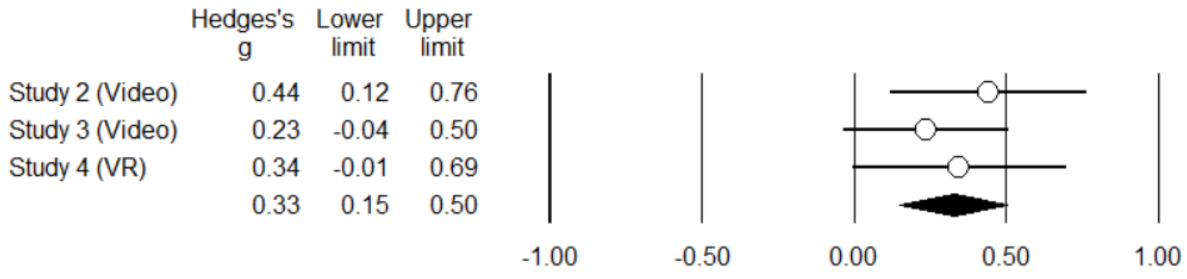

Attention Checks used through Studies 1-4

Study 1a (USA)

- This is an attention check, pick 2 (1 = *not at all*, 5 = *extremely*)
- This is an attention check, pick 5 (1 = totally disagree, 7 = totally agree)

Study 1b (Australia)

- This is an attention check, pick 5 (*1* = totally disagree, *7* = totally agree)
- This is an attention check, pick strongly disagree (1 = strongly disagree, 5 = strongly agree)
- This is an attention check, please pick green (red, yellow, blue, pink, green)

Study 2 (Australia)

- Attention checks were administered immediately after the video conditions.
- If in the non-nature awe condition:
    - Initially, what colour was the liquid at the bottom? (Pink, Lime, Orange, White)
- If in the negative nature awe condition:
    - What was the last scene of the video? (Volcano, Tsunami, Tornado, Bush fire)
- If in the control condition:
    - What was being built in the video? (Countertop, Bench, Door, Chair)

Study 3 (USA)

- Attention checks were administered immediately after the video conditions.
- If in the control condition:
    - What is the purpose of the pile of rocks? (trail marker, grave marker, danger sign, shrine for forest spirits)
- If in the awe condition:
    - In the last scene of the video, what was the camera flying towards? (River and mountain, A city, A sunset, A sunrise over an ocean)

Study 4 (VR – Australia)

- We reasoned that because of the interactive nature of the face-to-face lab setting and the short number of surveys, there was no need to administer attention checks in Study 4.

Supplementary References

Crimston, C. R., Bain, P. G., Hornsey, M. J., & Bastian, B. (2016). Moral expansiveness: Examining variability in the extension of the moral world. *Journal of Personality and Social Psychology, 111*(4), 636. https://doi.org/10.1037/pspp0000086

Hayes, A. F. (2009). Beyond Baron and Kenny: Statistical mediation analysis in the New Millennium. *Communication Monographs*, *76*(4), 408-420. https://doi.org/10.1080/03637750903310360

Hayes, A. F. (2018). *Introduction to mediation, moderation, and conditional process analysis: A regression-based approach* (2nd.ed.). The Guilford Press.
